# Ethanol exposure drives colon location specific cell composition changes in a normal colon crypt 3D organoid model

**DOI:** 10.1038/s41598-020-80240-1

**Published:** 2021-01-11

**Authors:** Matthew Devall, Sarah J. Plummer, Jennifer Bryant, Lucas T. Jennelle, Stephen Eaton, Christopher H. Dampier, Jeroen R. Huyghe, Ulrike Peters, Steven M. Powell, Graham Casey

**Affiliations:** 1 Department of Public Health Sciences, Center for Public Health Genomics, University of Virginia, Charlottesville, VA USA; 2 Department of Surgery, Center for Public Health Genomics, University of Virginia, Charlottesville, VA USA; 3 Digestive Health Center, University of Virginia, Charlottesville, VA USA; 4 Public Health Sciences Division, Fred Hutchinson Cancer Center Research Institute, Seattle, WA USA

**Keywords:** Computational biology and bioinformatics, Molecular biology, Gene expression, Genomics

## Abstract

Alcohol is a consistently identified risk factor for colon cancer. However, the molecular mechanism underlying its effect on normal colon crypt cells remains poorly understood. We employed RNA-sequencing to asses transcriptomic response to ethanol exposure (0.2% vol:vol) in 3D organoid lines derived from healthy colon (n = 34). Paired regression analysis identified 2,162 differentially expressed genes in response to ethanol. When stratified by colon location, a far greater number of differentially expressed genes were identified in organoids derived from the left versus right colon, many of which corresponded to cell-type specific markers. To test the hypothesis that the effects of ethanol treatment on colon organoid populations were in part due to differential cell composition, we incorporated external single cell RNA-sequencing data from normal colon biopsies to estimate cellular proportions following single cell deconvolution. We inferred cell-type-specific changes, and observed an increase in transit amplifying cells following ethanol exposure that was greater in organoids from the left than right colon, with a concomitant decrease in more differentiated cells. If this occurs in the colon following alcohol consumption, this would lead to an increased zone of cells in the lower crypt where conditions are optimal for cell division and the potential to develop mutations.

## Introduction

While a growing number of inherited genetic variants have been associated with colorectal cancer (CRC) risk in recent years^1,2^, environmental factors, such as alcohol consumption have also been strongly implicated^3^. In 2011, a large meta-analysis across a total of 61 cohorts found that moderate drinking (1–4 drinks per day) increased CRC risk by 21%. Further, heavy alcohol drinking (≥ 4 drinks per day) increased CRC risk by 52%^4^. Despite this, our knowledge of the molecular mechanism underlying the effect of alcohol consumption on normal epithelial cells of the colon crypt is poorly understood. Interrogation of the effect of alcohol exposure on colon-derived normal epithelial cells has the potential to provide important insight into CRC initiation and development.

While the colon is generally considered to be one organ, left and right colon display fundamental differences in vascular anatomy^5^ and gene expression in arising tumors^6^. Epidemiological studies have revealed location-dependent differences in CRC incidence with respect to alcohol consumption. For example, alcohol consumption has been associated with increased risk for cancers of the left colon and rectum in males^7^, while other studies have indicated increased risk for tumors of the left colon in regular alcohol drinkers across genders^8,9^. This implies that colon location should be a relevant factor to consider when assessing the effects of alcohol exposure in normal colon crypt epithelial cells.

The effect of ethanol exposure in relation to CRC risk has been examined previously by RNA-sequencing (RNA-seq) in CRC cell lines^10^, animal models^11^ and epidemiological studies using colon biopsies^12^. Each of these models have their challenges. In recent years, methods to establish and grow normal colon epithelial cell 3D organoid models have been developed^13^. This model reflects the cellular complexity of the stem cell niche of the colon crypts from which they were derived and represents a potentially important system for modelling environmental exposures. Recently, our group published a pilot investigation into the effect of ethanol exposure on gene expression and chromatin accessibility in normal colon organoids^14^. Despite the limited sample size, 1,965 differentially expressed genes (DEGs) were identified, a subset of which were also associated with differential chromatin accessibility. However, this study was limited to organoid lines derived from the right colon of three male subjects. Here, we extend our investigation to include a larger number of organoid lines derived from the left or right colon of both males and females.

In this study, we show that 3D organoids derived from the left versus right colon showed a striking differential response in the gene expression changes following ethanol exposure. We also observed profound differences in cellular composition in organoids derived from different colon locations. Ethanol exposure led to an increase in overall number of proliferative transit amplifying cells at the expense of differentiated cells, particularly in the left colon. This result has important implications for cancer risk as it suggests that alcohol consumption could lead to an increased zone of cells in the lower part of the crypt in the colon, where conditions are optimal for cell division and the potential to develop mutations. These results provide important insight into the molecular basis for the observed differential effect of alcohol consumption on risk for different locations of the colon.

## Results

### Ethanol exposure modestly affects cell growth and viability

Counts and viability measures of a subset of left colon organoid lines (n = 3) used in the full experiment were recorded before and after exposure to ethanol or water control. Ethanol exposure led to modest reductions in both cell viability (9.8%, *P* = 0.01) and proliferation (34.2%, *P* = 0.03). Similar reductions to cell viability (11.2% *P* = 0.03) and proliferation (14.8%, *P* = 0.01) were observed in a technical replicate of the same experiment (Supplementary Figure 1). Representative images of each of the three lines are shown (Supplementary Figure 2).

**Figure 1:**
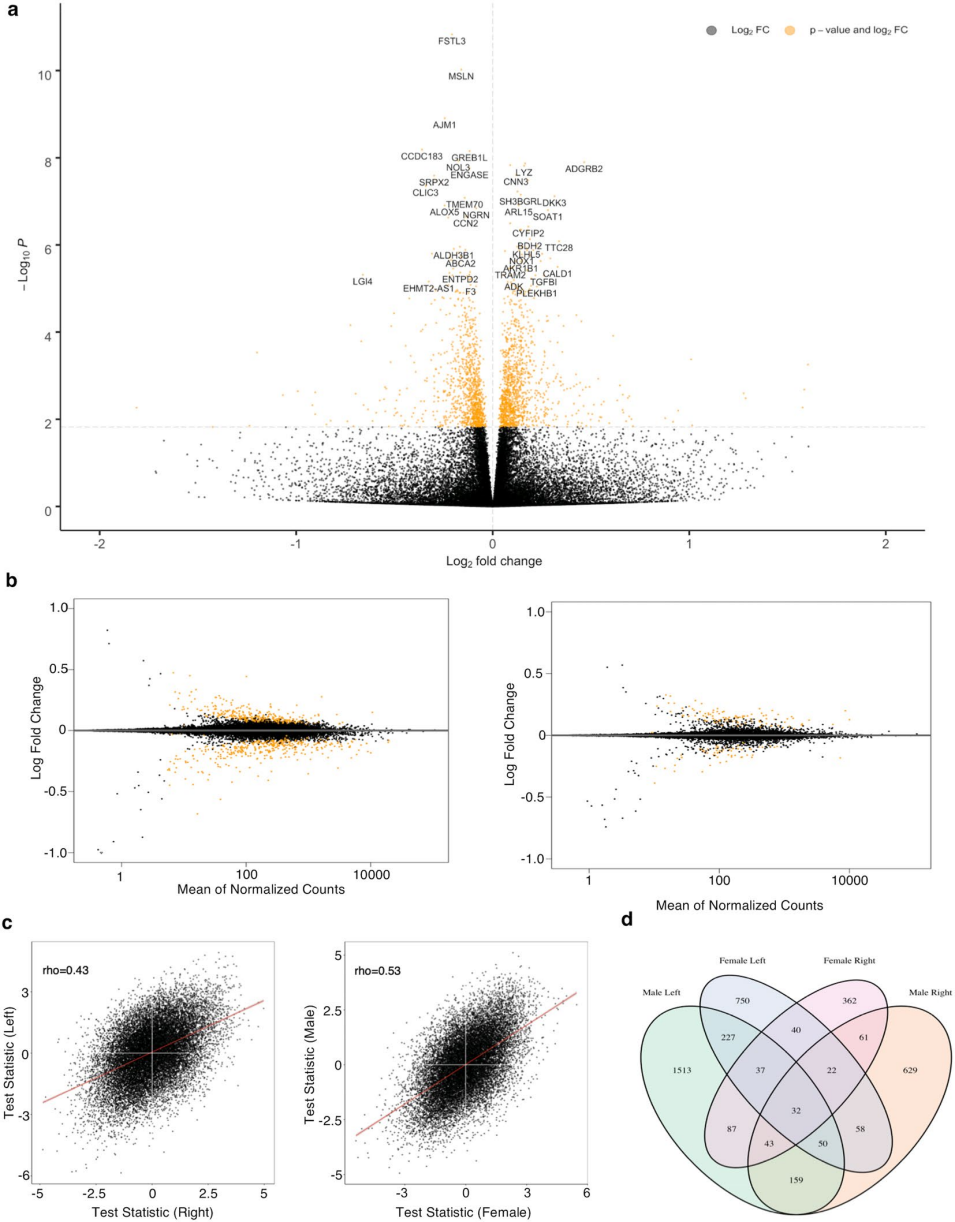
Summary of regression analysis for RNA-seq. (**A**) Volcano plot of DEGs. Positive log fold change corresponds to increased expression in ethanol-treated organoids. (**B**) MA plot highlighting differential expression in left colon (left panel) and right colon organoids (right panel) in response to ethanol. Orange values indicate significance. Positive log fold change corresponds to increased expression in vehicle control organoids. (**C**) Test-statistics generated from each colon location (left panel) and sex (right panel) subsets were correlated (spearman rank) to each other. (**D**) Stratified analysis by colon location and sex revealed that a greater number of nominal DEGs were identified in male, left colon organoids than any other subset.

**Figure 2:**
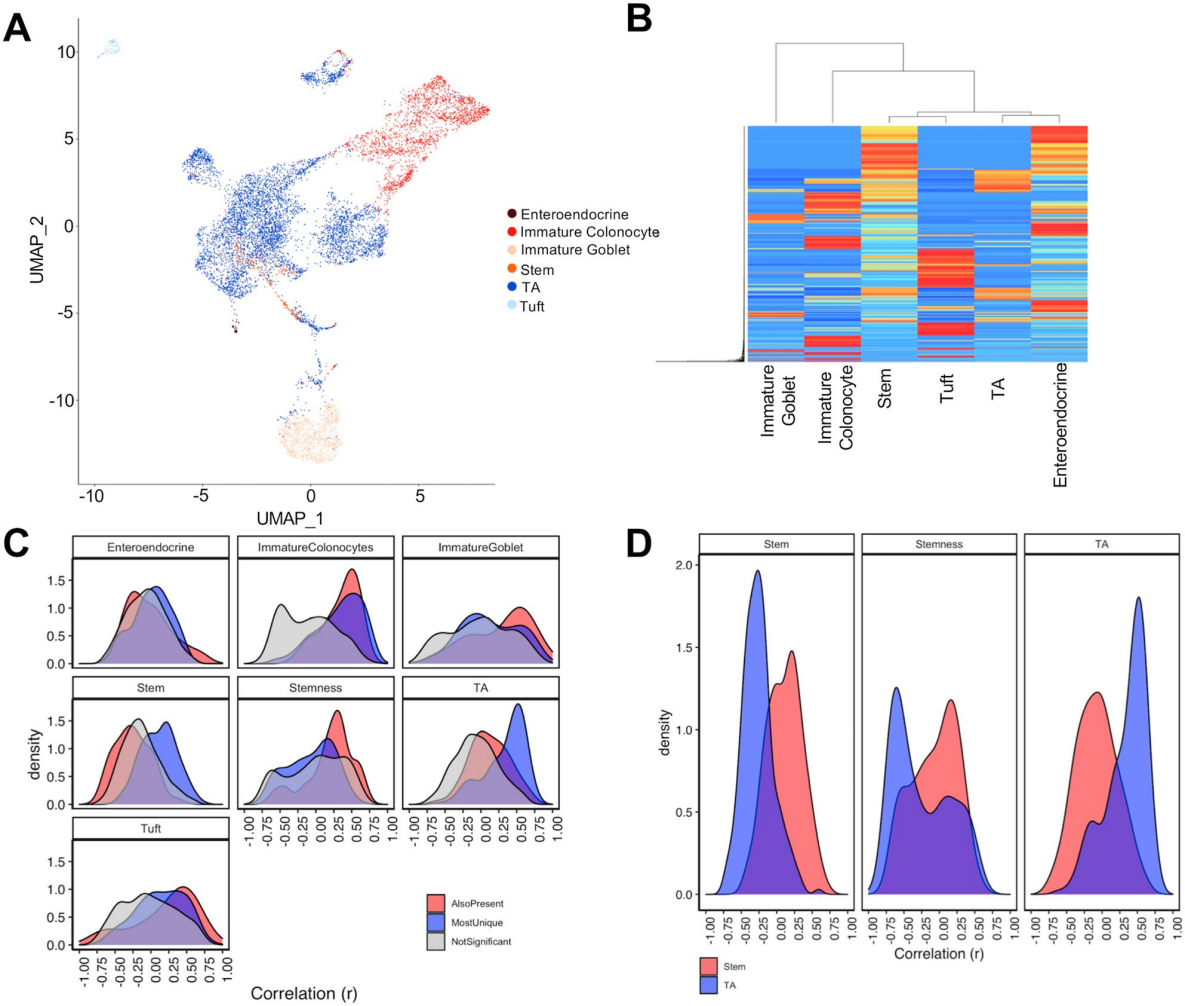
Cell composition analysis in ethanol treated colon organoids. (**A**) Uniform Manifold Approximation and Projection plot of 9659 single cells included in the analysis. (**B**) Heatmap of average gene expression of each gene present in the cell signature matrix. (**C**) Cell scores generated from Cibersortx were correlated to gene markers that were: most unique to that cell-type (blue); significant in that cell type (red); not significant in that cell type (grey). For comparison stemness index scores were compared to stem cell markers. (**D**) Markers of stem cells and TA cells were correlated to Cibersortx cell scores and the stemness index. A separation of markers for TA and stem cells may act as an indicator for performance of cell score in defining these cell-types.

### Substantial differences in transcriptomic responses were observed between 3D organoids derived from the left and right colon

Three-dimensional colon organoids were cultivated from either left (n = 16) or right (n = 18) normal colon biopsies of different individuals obtained following colonoscopy. Samples were excluded based upon previous or family history of CRC and/or biopsies derived from an individual with more than three polyps (Supplementary Table 1). These samples were excluded to reduce heterogeneity and to more precisely model the effects of ethanol exposure in the general population. Paired regression across all samples (n = 34 pairs) identified 2,162 significant DEGs following multiple testing correction (Fig. 1A). Consistent with a reduction in viability in ethanol treated organoids, we found that 14 of 87 previously identified apoptosis-related genes^15^ were differentially expressed in our analysis, of which 11 were upregulated following ethanol treatment (Supplementary Table 2). Pathway analysis revealed an enrichment for relevant pathways such as genes related to ethanol (q = 2.55E^−04^) and colonic neoplasms (q = 0.026) in DEGs that showed increased expression following ethanol treatment (Supplementary File 1). Of the 1,965 DEGs identified in our pilot study^14^, 749 (38.18%) were at least nominally significant (*P* = 0.05) in our larger sample set. To validate these changes, a subset of genes was selected for qPCR analysis in a subset of samples (n = 6). This subset included RNA from four left and two right colon-derived organoids. Highly significant genes were selected to be assayed if: they were novel; had been previously associated with alcohol treatment in tissues other than colon^16^; or previously associated with CRC through genome-wide association studies (GWAS)^1,17,18^. Of the 11 genes chosen for validation, seven were significant and followed the same direction of effect (Supplementary Table 3). We performed an analysis of location-specific responses to ethanol treatment by stratifying our dataset accordingly. We identified 4.26-fold greater number of significant DEGs in left versus right colon organoids (711 vs 167), despite similar sample sizes (Fig. 1B). Stratification by sex identified 548 and 124 significant DEGs in males and females respectively. Highly significant correlations were observed between test statistics generated from each analysis of location (rho = 0.43, *P* < 2.2E−16) and sex subsets (rho = 0.53, *P* < 2.2E−16), indicating moderate to strong overall concordance of transcriptomic response to ethanol (Fig. 1C). Stratification by colon location and sex revealed moderate overlap of nominally significant DEGs, while the greatest number of DEGs identified in organoids were derived from male left colon (Fig. 1D).

**Table 1:** Differentially expressed genes in ethanol treated organoids overlap markers of colon cell types. Number of genes surviving FDR are shown in brackets.

Direction	Colonocyte	Goblet	Enteroendocrine	Stem	TA	Tuft
**Ful**l						
Up	18 (13)	15 (9)	9 (5)	27 (18)	91 (89)	10 (8)
Down	67 (45)	40 (27)	3 (1)	3 (2)	16 (12)	15 (10)
**Left**						
Up	11 (4)	10 (1)	5 (1)	21 (6)	65 (18)	7 (2)
Down	57 (19)	37 (11)	2 (1)	4 (1)	11 (4)	14 (7)
**Right**						
Up	5 (0)	3 (0)	2 (0)	7 (0)	35 (0)	5 (0)
Down	16 (0)	9 (0)	0 (0)	3 (0)	13 (0)	4 (0)

**Table 2:** Regression analysis of absolute cell scores in ethanol treated organoids. Positive values indicate increased absolute cell score in vehicle control colon organoids.

	Immature Colonocyte	TA	Immature Goblet	Stem	Enteroendocrine	Tuft
**Ful**l						
Mean difference	1.25	− 1.47	0.84	0.19	0.13	0.12
95% Confidence Interval	1.00, 1.59	− 1.09, − 2.98	0.48, 1.16	0.89, 2.75	0.41, 1.07	0.01, 0.26
*P* value	0.02	0.01	1.54E^−03^	0.68	0.52	0.016
**Left**						
Mean difference	1.63	− 2.13	0.74	0.42	0.45	0.14
95% Confidence Interval	1.56, 2.61	− 1.95, − 4.39	0.61, 1.12	1.44, 4.74	0.64, 0.84	0.12, 0.16
*P* value	0.06	0.049	0.032	0.58	0.19	0.03
**Right**						
Mean difference	0.91	− 0.89	0.933	− 0.015	− 0.15	0.11
95% Confidence Interval	1.31, 1.83	− 1.11, − 4.06	0.734, 1.972	1.114, 3.108	− 0.42, − 1.79	0.15, 0.41
P value	0.20	0.14	0.023	0.980	0.57	0.18

**Table 3:** Summary results of enrichment analysis from one-way Fisher’s exact test of overlap between significant DEGs identified in regression on cell score and known markers of cell composition.

	Immature Colonocyte	TA	Immature Goblet	Stem	Enteroendocrine	Tuft
Odds ratio	12.55	3.96	1.47	1.67	1.56	1.49
*P* value	< 2.2E^−16^	< 2.2E−16	8.12E^−03^	0.03	0.097	0.04

### Analysis of cell-type specific markers implied differences in cellular composition of 3D colon organoids of left versus right colon

We examined the overlap between DEGs identified in our initial paired regression of all subjects with a list of cell-type-specific gene markers generated from previously published scRNA-seq data from normal colon biopsies^19^. Of the 2,162 significant DEGs associated with ethanol treatment, 58 were potential gene markers of colonocytes^19^. The expression of 45 of these DEGs was reduced in ethanol treated organoids. A similar pattern was identified in goblet cells. However, significant DEGs that marked enteroendocrine cells, stem cells and TA cells were increased in ethanol treated organoids (Table 1). This suggests a shift in cell population following ethanol treatment towards increased stem and TA cells. This effect appeared greater in left than right colon organoids (Table 1).

### Differential cell composition changes between organoids of the left versus right colon in response to ethanol were confirmed following application of a single cell deconvolution framework for bulk RNA-Seq data

To test the hypothesis that the effects of ethanol treatment on colon organoid populations were in part due to differential cell composition, we performed single cell deconvolution analysis of a publicly available RNA-seq dataset of normal colon. We downloaded, filtered and processed publicly available, single cell RNA-seq (scRNA-seq) data using Seurat^20^. Our final dataset consisted of 9,659 cells across six cell populations (Fig. 2A). A cell signature matrix was generated using Cibersortx^21^ and hierarchical clustering of these genes was used to separate cell populations (Fig. 2B). Absolute cell composition scores were generated for each sample. Extending upon earlier findings, total absolute scores were reduced in ethanol treated samples across the full dataset (*P* = 0.03). Paired regression of absolute cell scores between treatment and vehicle control identified significant differences in cell composition in response to ethanol. These differences confirm cellular composition changes predicted by gene expression analysis of known marker genes. These effects were more pronounced in organoids of the left colon (Table 2). Taken together, this indicates that exposure to ethanol affected both, the overall number of cells and the cellular composition of the organoid population, with an expansion of TA cells and a restriction in differentiated lineages.

We performed a regression analysis of vehicle control (water) colon organoids to quantify the relationship between gene expression and individual cell composition scores. Fisher’s exact test identified enrichments for known cell markers in five of six significant DEG lists, indicating that cell scores accurately captured cellular composition changes in this model (Table 3). Significant marker genes for each cell type were correlated to cell composition scores. The stemness index has previously been generated to estimate the extent of cellular differentiation of cancer tumors^22^. For comparison, we estimated the stemness index score of colon organoids and correlated scores to expression of relevant genes (Fig. 2C). Stem cell scores generated in the current approach appeared better able to capture stem cell gene expression (Fig. 2D).

### Identification of cell-type agnostic gene expression differences in ethanol treated colon organoids

**Figure 3:**
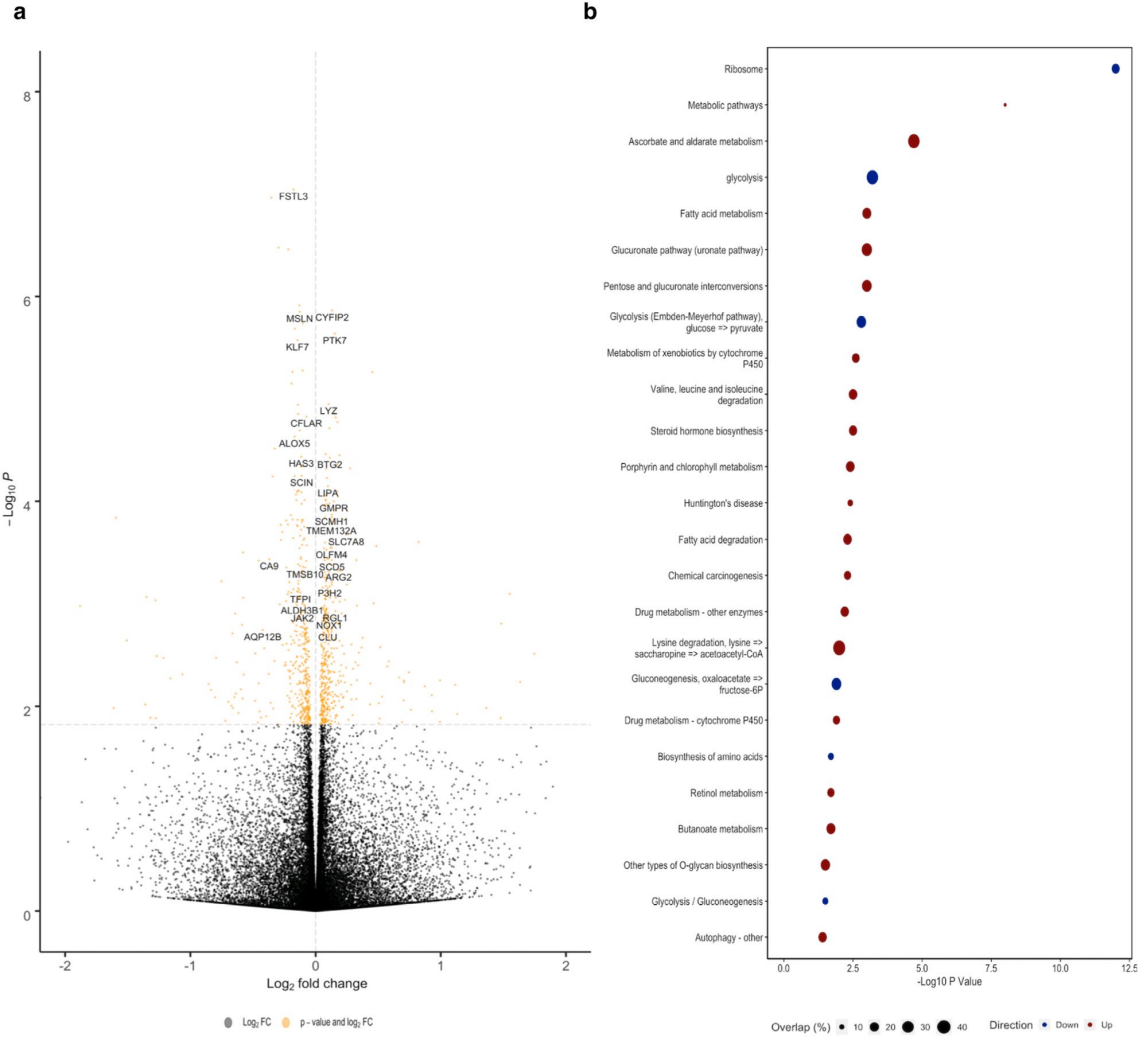
Summary of regression for cell-type agnostic approach. (**A**) Volcano plot of DEGs identified after adjusting for cell composition. Positive log fold change corresponds to increased expression in ethanol-treated organoids. (**B**) Results from pathway enrichment analysis of genes found to be nominally overexpressed (red) and underexpressed (blue) following ethanol treatment. Overlap indicated the percentage of DEGs found in gene list that comprise that pathway.

Cell composition scores were incorporated as covariates and regression analyses were performed in the full dataset (herein referred to as the adjusted model), as well as in stratified analysis of sex and colon location. Stratification by colon location identified 68 and three significant DEGs in left and right colon organoids, respectively, after adjustment for cell composition (Supplementary Table 4). Surprisingly, stratification of the dataset by sex revealed more DEGs in females than males following adjustment for cell composition (26 vs 12; Supplementary Table 5). In an analysis of all colon organoid pairs, we identified 271 significant cell-type agnostic DEGs after multiple testing corrections. Of these, 216 were also significant in our unadjusted, full model, while 55 were novel (Fig. 3A, Supplementary Table 6). All genes validated by qPCR remained significant in the adjusted analysis. RB Binding Protein 7, Chromatin Remodeling Factor (*RBBP7*)*,* Secretogranin V (*SCG5*) and DNA Polymerase Delta 3, Accessory Subunit (*POLD3*), which were not validated by qPCR, were no longer significant upon adjusting for cell composition. However, a significant increase in Aldehyde Dehydrogenase 7 Family Member A1 (*ALDH7A1*) was still seen (q = 0.02). Of the 55 novel DEGs, 40 mapped to genes present in a microarray analysis of ethanol exposure in blood^23^, 15 of which were significant with the same direction of effect (Supplementary Table 6).

Analysis of KEGG pathways identified a consistent downregulation of genes in energy metabolism pathways, while an upregulation of many drug-related processes could also be observed in response to ethanol treatment (Fig. 3B). Pathway analysis across other databases^24^. Among nominally overexpressed DEGs, we identified greater enrichment for ethanol (q = 1.97E^−07^) and colonic neoplasms (q = 2.29E^−03^) than we observed in our unadjusted analysis. Further, we identified several more alcohol-related enrichments in our adjusted analysis, such as alcohol biosynthetic process (q = 1.11E^−03^) and secondary alcohol biosynthetic process (q = 6.17E^−03^), which were not identified in the original model. Taken together, these findings imply that controlling for cell composition in our dataset increased specificity towards the identification of ethanol-related genes. Finally, we also identified significant enrichments for pathways relevant to crypt stem cell niche biology, such as positive regulation of canonical Wnt signaling (q = 6.28E^−03^), in our overexpressed DEGs.

To attribute potential functional relevance to these DEGs, paired regression analysis was performed on CRC data downloaded from The Cancer Genome Atlas (TCGA)^25^. Regression analysis was performed on normal adjacent tissue and tumor pairs after controlling for variation attributed to differences in stemness^22^. Of the FDR corrected DEGs identified following adjustment for cell composition in ethanol treated organoids, 270 were expressed in TCGA-colon adenocarcinoma. Of these, 138 were found to be significant (Supplementary Table 7). One-way Fisher’s exact test revealed that cell-type agnostic ethanol-treated DEGs were significantly enriched for CRC-related DEGs (*P* = 1.01E^−07^)^16^.

### Inference of cell-type specific responses to ethanol treatment

Single cell deconvolution has been used to infer cell-type-specific expression quantitative trait loci from bulk RNA-seq data^26^. We adapted this strategy for use in our colon organoid dataset to examine cell-type-specific responses to ethanol exposure. Cell-type-specific responses were examined combining data from left and right organoids. For genes present in more than one cell-type, only the most significant interactions are reported, but notable exceptions are discussed. We identified 3,212 significant DEGs in stem cells, 1,064 in TA cells, 552 in immature goblet cells, 160 in tuft cells, and 415 in enteroendocrine cells. No gene survived multiple testing corrections in immature colonocytes (Supplementary File 2).

In the stem cell-specific analysis, we highlight three major findings: altered cell cycle gene expression; a reduced expression of genes involved in DNA repair; and a reduction of stem cell-specific markers. Of the genes most significantly altered in stem cells, many were involved in cell cycle regulation, and were decreased in ethanol treated organoids. Pathway analysis of DEGs significantly reduced following ethanol exposure revealed enrichment for multiple cell cycle processes, suggesting that ethanol treatment may lead to cell cycle delay or arrest in colon stem cells. This analysis also identified a strong enrichment for the DNA repair pathway (q = 1.26E^−42^) and included reduced expression of MutL Homolog 1 (*MLH1*; q = 6.88E^−03^)*,* MutS Homolog 2 (*MSH2*; q = 2.77E^−03^), and *MSH6* (q = 3.88E^−04^). These genes are involved in mismatch repair, a process defective in microsatellite instable tumors and Lynch Syndrome^27^. Finally, we identified reduced expression for markers of stem cells including Leucine Rich Repeat Containing G Protein-Coupled Receptor 5 (*LGR5*; q = 8.51E^−04^), Mex-3 RNA Binding Family Member A (*MEX3A*; q = 0.017), and Achaete-scute Complex Homolog 2 (*ASCL2*; q = 6.54E^−03^).

Multiple, overlapping pathways and DEGs were observed between TA and stem cell populations, the two major proliferating cell-types identified in colon organoids. Often, these similarities were found to have opposite directions of effect. While we observed a significant decrease in *LGR5* expression in the stem cell population exposed to ethanol, a less significant increased expression was observed in the TA cell population (q = 0.071). TA cell-specific overexpressed DEGs were also enriched for multiple overlapping processes including: cell cycle (q = 1.01E^−07^) and DNA repair (q = 2.12E^−09^), showing an opposing effect following ethanol exposure in the TA cell population compared to stem cells (Supplementary File 2).

Recently, bulk and scRNA-seq analysis for enteroendocrine cell lineage tracing revealed multiple time-point specific transcriptional regulators involved in coordination of enteroendocrine differentiation in the small intestine^28^. We were interested in determining whether ethanol specifically affected expression of these regulators. For this analysis, all enteroendocrine cell results were considered. Interestingly, regulators of early enteroendocrine cells were typically reduced following ethanol treatment, while the inverse appears to be true for later cell stage regulators (Supplementary File 2 – Enteroendocrine Regulators). This may indicate a shift in population maturity of enteroendocrine cells during ethanol treatment.

## Discussion

In this study, we measured the effect of ethanol exposure on global gene expression profiles of colon organoids derived from normal colon crypts, and identified 2,162 significant DEGs. When stratified by colon location, we observed a differential transcriptomic response that was far greater in organoids derived from the left versus right colon. We performed single-cell deconvolution^21^ to both quantify and adjust for the effects of cell composition in our bulk RNA-seq dataset. Following adjustment for cell composition, organoids from the left colon still displayed a greater transcriptomic response to ethanol treatment. This result is consistent with the observation from epidemiological studies of an association between alcohol and increased risk of developing left CRC tumors^8,9^. It is also notable that recent studies have revealed important global gene expression differences in tumors arising from different colon locations^6^, possibly reflecting not only differential cell composition of crypt epithelial cells of left versus right colon, but also differential site-specific responses to epidemiological risk factors such as alcohol. The difference in number of DEGs between males and females observed following stratification by sex in our unadjusted model were not maintained when we considered the effects of cell composition. This may indicate that original results were confounded by cell composition variation and highlights the importance of adjusting for such effects in the organoid system.

Our additional paired analysis of TCGA-colon adenocarcinoma RNA-Seq data revealed that the significant gene expression differences were enriched for CRC-related genes. Indeed, pathway analysis of overexpressed DEGs in our adjusted full model revealed an enrichment for genes found to positively regulate the canonical Wnt signaling pathway, which is not only required for stem cell homeostasis and proliferation of progenitors, but is also dysregulated in CRC^29^. Cell composition analysis identified a significant increase in the overall TA cell population following ethanol treatment. When stratifying our dataset, we found that this increase was only significant in left colon organoids. A similar pattern was observed in tuft cells, which were only significantly reduced in left colon organoids following exposure to ethanol, while immature goblet cells were consistently decreased in both datasets. Consumption of alcohol has previously been shown to reduce goblet cell content in rectal samples, a reduction which was ameliorated following a two week period of abstinence^30^. This effect was also observed in murine models of intestinal epithelia^31^. A reduction in goblet cells not only disrupts protective mucus production, but may also may lead to alterations in the gut microbiome and increased gut leakiness^31^. Increased cell proliferation has been reported in rectal cells of human alcohol abusers^32^ as well as in both small and large intestinal epithelium of alcohol-fed mice^33^. Activation of the canonical Wnt signaling pathway has been shown to correspond with reduced goblet but not enteroendocrine cell numbers^34^. Activation of β-catenin has also been shown to correlate with a reduction of colonocyte cells and the establishment of a crypt progenitor cell abundant phenotype in CRC^35^. We hypothesize that this increase in Wnt signaling leads to an increased TA cell population and an overall reduction in numbers of both goblet cells and colonocyte cell populations, either through their ablation, or through a lack of TA cell differentiation. That there is a more significant increase in the TA cell population following ethanol exposure in left colon organoids highlights one plausible explanation for the increased risk of distal CRC in alcohol drinkers. However, other factors may also play a role in this shift in cellular composition. For example, it is unclear if cell competition^36^ plays an active role in the expansion of TA cells at the expense of more differentiated cells, and whether this is more relevant in organoids derived from the left versus right colon. Determining the mechanism through which TA cells expand at the expense of more differentiated cell populations may improve our understanding of how alcohol consumption increases CRC risk. Sustained cell division of TA cells has been shown to lead to pathologies such as the establishment of colonic crypt hyperplasia, an important feature of ulcerative colitis^37^. Further studies that seek to understand the trajectory of these changes over time should provide much improved understanding of the role of ethanol in the colonic crypt.

We extended our analytical approach of cellular deconvolution to identify cell-type-specific responses to ethanol exposure. We note that while cell cycle pathways are enriched in downregulated stem cell DEGs, they are upregulated in TA cells. The reduced expression of cell cycle genes by ethanol exposure in stem cells has been previously observed in neural stem cells^38^. Ethanol exposure also leads to reduced neural stem cell proliferation in rat brain, but leads to an increase in stem cell progenitors^38,39^, similar to our study. Acetaldehyde is a toxic by-product of alcohol metabolism that leads to DNA crosslinking, DNA double-stranded breaks and chromosomal rearrangement in hematopoietic stem cells^40^. The identification of an enrichment for downregulated genes involved in DNA repair and in chromosome organization in colon stem cells exposed to ethanol may be a potential mechanism by which alcohol increases CRC risk. Alcohol may contribute to DNA damage in stem cells by increasing reactive oxygen species (ROS)^41^. Increased ROS has been consistently associated with alcohol in multiple cell-types and experimental designs^41^. We observed that a number of pathways related to oxidative stress were enriched in stem-cell specific DEGs overexpressed following ethanol treatment. Balancing ROS levels is critical for the regulation of stem cell differentiation and self-renewal^42^. Thus, an alcohol-induced increased elevation of ROS levels may not only lead to DNA damage, but over extended periods, may also lead to a reduction in the self-renewal capacity of healthy intestinal stem cells.

Adopting an approach that integrates cell-type-specific responses to ethanol in our study helped to better elucidate the contradictions with regards to cell-type-specific gene expression changes. For example, our primary analysis of all subjects revealed an overall increase in the expression of *LGR5*. However, cell-type-specific analyses revealed *LGR5* to be decreased in the stem cell population following ethanol exposure, whereas *LGR5* was increased in the TA cell population, which likely represents the majority cell population within colon organoids. This highlights the importance of a cell-type-specific approach. These differential cell-type-specific effects require further investigation.

There are several limitations to our study, which broadly fall into one of three categories: bioinformatic, experimental and organoid-specific. For experimental limitations, we chose a single time-point and dose, given the size of the organoid study. This dose aimed to reflect circulating blood levels of alcohol during the daily, regular drinking of alcoholics^43^. Dose-and time-dependent effects of alcohol have been observed with regard to CRC risk^4^ and gene expression in CRC cell lines^44^. This dose (43 mM, 2–4 drinks) has previously been shown to approximate with blood alcohol levels determined through in vivo studies of rats in models of alcohol treatment^45^. It has previously been chosen for studying the effects of alcohol treatment in monolayer cultures of colon epithelial cells^43,45^ and small intestinal organoids^46^. However, relating dosage in the blood to the dose colon epithelial cells are exposed to in vivo can be problematic. Large-scale pharmacodynamic studies of alcohol absorption within the colonic epithelium should be considered in the future to improve the modelling of ethanol treatment in vitro*.* Future studies should be extended to include multiple time-points and longer time frames to better capture dynamic changes of cell composition. We also note that alcohol metabolites such as acetaldehyde may have independent or synergistic effects on gene expression that cannot be delineated through our approach. Finally, while a 72-h ethanol exposure is unlikely to drive colon organoids to a tumorigenic state, we identify some gene expression changes similar to those seen in CRC tumors. Whether the differential expression we observed in our single time point study persists and acts to drive carcinogenesis is unknown. With regards to bioinformatic limitations, mature tuft cell and enteroendocrine cell populations from scRNA-seq data were used to generate our cell signature matrix for deconvolution and we note that this may affect the interpretation of results. Given the nature of the organoid model, it is unlikely that these populations have reached maturity in organoids and may instead be present in a progenitor state. However, late-stage enteroendocrine cell regulators identified in a scRNA-seq study of small intestine enteroendocrine cells^28^ were generally overexpressed in our enteroendocrine cell-specific analysis, while early stage regulators were reduced in ethanol treated enteroendocrine cells. This may indicate that a more mature enteroendocrine cell population is present following ethanol treatment. Further, the original deconvolution approach demonstrated that mouse scRNA-seq data could be used to estimate cell composition in human bulk RNA-seq, highlighting the robustness of the method^26^. Single-cell deconvolution is a developing field that provides high-resolution modelling of bulk RNA-seq datasets. It overcomes limitations of scRNA-seq, such as high cost, which often limits sample sizes used and as a result, reduces generalizability of findings. However, future studies should also incorporate independent validation methods such as immunostaining or some additional single-cell RNA-seq. With regards to the colon organoid model system, findings reported here need to be considered within the context of the normal cells of the colon where a number of differences exist between the colon organoid system and the environment of the colonic crypt. For example, the colon mucosal barrier forms a protective layer against numerous insults. Increasing the permeability of intestinal mucosa is one method by which alcohol has been proposed to cause dysfunction^47^. Colon organoids do not possess a mucosal barrier, as such, these effects cannot be appropriately modelled within this system. Further, studies have consistently highlighted the important role of LGR5^+^ stem cells in colon carcinogenesis^48^. However, we recognize that the tumor microenvironment also plays an important role in the onset and development of CRC, one which may be influenced by alcohol^49^. While some attempts have been made to co-culture intestinal organoid models and the tumor microenvironment^50^ or overlay immune cell contexts^51^, these methods have some limitations^51^. Nevertheless, these co-culture methods should be considered in future experimental designs.

These data show that ethanol exposure leads to an overall increase of proliferating TA cells and a concomitant reduction in more differentiated cell types in organoids derived from the left colon, and to a lesser extent in organoids derived from the right colon. This accumulation in TA cells may occur in response to an upregulation of WNT signaling. If this occurs in colon crypts following alcohol intake, it would result in an increased zone of cells in the lower crypt where conditions are optimal for cell division and the potential to develop mutations. These in turn, may lead to an increased likelihood of developing premalignant conditions such as polyp growth and crypt hyperplasia. These findings reveal a potential mechanistic basis for the observed alcohol-related increase in risk for left sided colon cancer.

## Methods

All authors had access to the study data and had reviewed and approved the final manuscript.

### Subject population and demographics

Subjects scheduled for screening or surveillance colonoscopy were enrolled under an approved Institutional Research Board (IRB) protocol of the University of Virginia (UVA) after providing informed consent through the period of July 2017–March 2019. For each individual, left and right colon mucosa biopsies were collected separately following colonoscopy using standard forceps. Biopsies were obtained immediately distal to the hepatic flexure (right colon) or immediately distal to the splenic flexure (left colon). All subjects presented with an absence of personal or family history of CRC, and the majority of subjects had no history of polyps. Only subjects of Caucasian descent were included in the study, to reduce heterogeneity. The experiments described were conducted under a UVA-approved IRB protocol. All methods were performed in accordance with the relevant guidelines and regulations, and were consistent with those required by both the NIH and UVA.

### Normal 3D colon organoid cell line growth and exposure

Normal 3D colon organoids included in the study were developed from biopsies of either left (n = 16) or right (n = 18) colon of different individuals using a modification of the method described by Sato et al.^13^. After isolation, colon crypts were embedded in Matrigel, and cell lines were established in growth media that included: advanced DMEM/F-12, 100U/ml penicillin, 100 μg/ml streptomycin, 10 mM Hepes, 1x N2, 1x B27, 2 mM GlutaMAX, 1 mM N-acetyl-cysteine, 10 mM gastrin, 50% L-WRN conditioned media, 500 nM A83-01, 10uM SB202190, 10 mM nicotinamide, 50 ng/ml EGF, 10 μM Y27632. Colon organoids were grown and passaged as needed (typically every 3–5 days) in 48-well culture plates. For routine passaging, organoids were gently dissociated to break down larger organoids, and to allow for the addition of fresh Matrigel and the removal of cellular debris. Briefly, Matrigel containing organoids was removed using a 1000μL pipette tip, suspended in 500μL wash medium and transferred to an Eppendorf tube. The organoid suspension was centrifuged at 300 × g for 5 min (4 °C) and the majority of supernatant was removed. The organoid pellet was resuspended in 1 mL TrypLE Express (Gibco, #12,604,013) and incubated at 37 °C for 10 min. 2 mL wash medium was added and organoids were dispersed using a 25G needle and 1 mL syringe by slowly passing them through the needle 6–8 times. Following centrifugation and removal of excess supernatant, the resulting cell pellet was resuspended in an appropriate volume of Matrigel to seed 30μL per well at a 1:3 split ratio. Plates were incubated at 37 °C for 15 min to polymerize Matrigel and then 500μL growth media was added. For this experiment, cells were treated with fresh growth media plus ethanol (Sigma Aldrich; Cat No: E7023) (2 μl per 1 ml of growth media), or fresh growth media plus 2 μl cell culture grade distilled water as a control. Media (with or without ethanol) was replaced every 24 h for 3 days in order to replace evaporating ethanol. Organoid lines used in the experiment were at passage 5–13. Ethanol dose and exposure time were chosen following a thorough literature search that identified similar ethanol treatment protocols, and aimed to model circulating blood levels of alcohol during daily, regular drinking of alcoholics^43^. Similar doses have been previously reported^10,14,43,46,52^.

### Cell viability and proliferation analysis

To determine the effect of ethanol treatment on cell viability and proliferation, three organoid cell lines were plated in duplicate in 48-well culture plates (approximately 0.25 ✕ 10^6^ cells per well), and fresh growth media was added. Organoids were then treated with ethanol, or vehicle-control, as described above. Following 72-h ethanol treatment, intact organoids were removed using a 1000μL pipette tip in 500μL DPBS and transferred to an Eppendorf tube. To dissociate organoids into single cells, the organoid suspension was centrifuged at 300 × g for 5 min (4 °C) and the majority of supernatant removed. The organoid pellet was resuspended in 1 mL Accutase (Corning, #25–058-CL) + 10 μM Y27632 and incubated at 37 °C for 20 min. 2 mL of wash medium was added and organoids were dispersed using a 25G needle and 1 mL syringe by slowly passing them through the needle 6–8 times. The suspension was again centrifuged and the pellet was resuspended in 1 mL TrypLE Express + 10 μM Y27632 and incubated at 37 °C for an additional 20 min. 2 mL wash medium was added and organoids were dispersed 6–8 times using a 25G needle and 1 mL syringe. Dissociated cells were centrifuged and then resuspended in 1 mL wash medium for cell count. This experiment was performed in duplicate. For each line, two counts were taken and the results were averaged together to obtain the final cell count and viability measure. A technical replicate of this experiment was then carried out. Cell counts were recorded using a Countess II Automated Cell Counter (Invitrogen: AMQAX1000), according to the manufacturer’s protocol. This instrument uses trypan blue staining combined with an auto-focus mechanism and image analysis. Two days after plating, cells were exposed with media containing ethanol or water as previously described. At the end of the 72-h treatment, cells were counted and viability determined. A mixed effects model was used to determine the effect of treatment status on organoid proliferation and viability. Images were also collected pre- and post- ethanol treatment at 10 × magnification using a bright field microscope.

### RNA isolation and sequencing

For RNA isolation, media was gently removed and 200 μl RNA lysis Solution RA1 with TCEP (Machery-Nagel; Cat No: 740902.50) was added to each well. The lysate was collected in a 1.5 ml Eppendorf tube. Each tube was vortexed vigorously and placed on ice. Further steps were carried out according to manufacturer’s protocol of the Machel-Nagel RNA XS kit. RNA was checked for quality using the Agilent 4200 Tapestation. Samples with RIN >  = 8.5 were considered for library preparation and sequencing using the Illumina NovaSeq6000 (Northwest Genomics Center at the University of Washington, Seattle). After sequencing, 100 bp paired-end reads were mapped to the human GENCODE transcriptome (release 29) using RSEM with STAR^53,54^ following quality control and marking of duplicates. An average of 70.41% reads uniquely mapped to the reference transcriptome, leaving an average of 64.08 million uniquely mapped reads per sample. Downstream analysis was carried out using R version 3.6.2^55^. Differential expression (DE) analysis was performed using DESeq2 after raw counts were imported using tximport^56,57^. For reporting, genes that survived Benjamani-Hochberg correction (BH = 0.1) were termed significant. Nominally significant (p = 0.05) genes were reported as such. For pathway analysis, gene lists were imported into ToppFun^24^. BH correction was set at 5% to reduce type I errors. Transcripts per million (TPM) were generated from the output of RSEM/STAR for additional analysis. Volcano plots were generated using the EnhancedVolcano package in R^58^.

### Quantitative PCR design and analysis

Gene expression validation experiments were performed using RNA isolated from an independent subset of the same colon organoid lines under the same experimental conditions. RNA for quantitative PCR (qPCR) was isolated as described above. A minimum of 800 ng of total RNA was reverse transcribed to first-strand cDNA using the High-Capacity cDNA Reverse Transcription Kit (Thermo Fisher). Pre-Designed TaqMan gene expression assays (Thermo Fisher) were used for quantification of select gene targets. Hypoxanthine Phosphoribosyltransferase 1 was selected as a control gene due to its moderate level of expression in these cells, and because we previously found that it was not affected by ethanol treatment^14^. Delta-CT values were then used as input for a paired eBayes regression in limma^59^.

### Single cell RNA-Seq cellular deconvolution

For single cell deconvolution experiments, we downloaded raw counts from a previously published scRNA-seq dataset of human colon biopsies^19^. Only cells from normal colon epithelium, containing greater than 250 unique genes and mitochondrial content < 15% were considered for further analysis in Seurat^20^. Visualization of cells considered for analysis was performed in Seurat. scTransform was applied to remaining cells, while regressing out mitochondrial percentage. Uniform manifold approximation and projection (UMAP) was performed, computing 50 PCs and setting findneighbors to 50. These adjustments were only used for visualization, in order to determine whether the subset of selected cell populations were distinguishable for further analysis. TPMs were generated for bulk and scRNA-seq data. Original cell types were used for cell labeling, and data was uploaded to Cibersortx^21^. M cells were removed due to low cell numbers. Mature goblet cells and colonocytes, colonocyte progenitors, cycling transit amplifying (cycling TA), and secretory TA populations were removed as delineation of similar cell types proved challenging for cell score estimation. Sub-populations of immature colonocytes were grouped, as were remaining TA cells. Mature tuft cells and enteroendocrine cells were included as no progenitor population was present in scRNA, leaving 9,659 cells across six cell populations. A signature matrix of genes able to best separate scRNA-seq defined cell populations was generated with the following parameters: expression cutoff = 0.15; q = 0.001; sampling = 1; gene range = 100–600; quantile normalization = disabled; permutations = 500. Cell scores were centered about the mean and scaled. For stratified analysis, this was performed on individual subsets independently. Scores were then imported as covariates into two regression models in DESeq2^56^:

1. Expression ~ Pair + Cell Proportions + Treatment
2. Full =  ~ Pair + Cell Proportion + Treatment + Treatment*Cell Proportion, Reduced =  ~ Pair + Cell Proportion + Treatment

Model 1 aimed to control for the effects of cell composition. Model 2 aimed to interpret cell-specific effects of ethanol exposure. For Model 2, a likelihood ratio test between the full and reduced model was performed to measure the effects of ethanol exposure on gene expression within a given cell type of interest.

### Analysis of external datasets

Publicly available data CRC HT-Seq-count data was downloaded from The Cancer Genome Atlas (TCGA)^25^ using the R package TCGAbiolinks^60^. Data was filtered to only include tumor samples with paired normal adjacent tissue and for which stemness indexes^22^ had been generated (n = 35 pairs). Paired regression was performed in DESeq2 while incorporating stemness index to aid in the control of cellular heterogeneity. For consistency with our previous analysis, genes were significant if they survived FDR correction (q < 0.1). To identify enrichment between analysis, one-way Fisher exact tests were performed on significant DEGs.

## Supplementary Information

Supplementary Information.

Supplementary Information.

Supplementary Information.

## References

[1] Huyghe JR (2019). Discovery of common and rare genetic risk variants for colorectal cancer. Nat. Genet..

[2] Law PJ (2019). Association analyses identify 31 new risk loci for colorectal cancer susceptibility. Nat. Commun..

[3] Rossi M (2018). Colorectal cancer and alcohol consumption-populations to molecules. Cancers.

[4] Fedirko V (2011). Alcohol drinking and colorectal cancer risk: an overall and dose-response meta-analysis of published studies. Ann. Oncol..

[5] Mike M, Kano N (2013). Reappraisal of the vascular anatomy of the colon and consequences for the definition of surgical resection. Dig. Surg..

[6] Maus MK (2015). Distinct gene expression profiles of proximal and distal colorectal cancer: implications for cytotoxic and targeted therapy. Pharmacog. J..

[7] Ferrari P (2007). Lifetime and baseline alcohol intake and risk of colon and rectal cancers in the European prospective investigation into cancer and nutrition (EPIC). Int. J. Cancer.

[8] Akhter M (2007). Alcohol consumption is associated with an increased risk of distal colon and rectal cancer in Japanese men: The Miyagi Cohort Study. Eur. J. Cancer.

[9] Zisman AL (2006). Associations between the age at diagnosis and location of colorectal cancer and the use of alcohol and tobacco (vol 166, pg 629, 2006). Arch. Internal Med..

[10] Moon JW (2014). Alcohol induces cell proliferation via hypermethylation of ADHFE1 in colorectal cancer cells. BMC Cancer.

[11] Barr T (2018). Concurrent gut transcriptome and microbiota profiling following chronic ethanol consumption in nonhuman primates. Gut Microbes.

[12] Slattery ML, Pellatt DF, Mullany LE, Wolff RK (2015). Differential gene expression in colon tissue associated with diet, lifestyle, and related oxidative stress. PLoS ONE.

[13] Sato T (2011). Long-term expansion of epithelial organoids from human colon, adenoma, adenocarcinoma, and Barrett's epithelium. Gastroenterology.

[14] Devall M (2020). Modeling the effect of prolonged ethanol exposure on global gene expression and chromatin accessibility in normal 3D colon organoids. PLoS ONE.

[15] Alcala S (2008). A high-throughput screening for mammalian cell death effectors identifies the mitochondrial phosphate carrier as a regulator of cytochrome c release. Oncogene.

[16] Davis AP (2019). The comparative toxicogenomics database: update 2019. Nucleic Acids Res.

[17] Lu Y (2019). Large-scale genome-wide association study of east asians identifies loci associated with risk for colorectal cancer. Gastroenterology.

[18] Dunlop MG (2012). Common variation near CDKN1A, POLD3 and SHROOM2 influences colorectal cancer risk. Nat. Genet..

[19] Smillie CS (2019). Intra- and inter-cellular rewiring of the human colon during ulcerative colitis. Cell.

[20] Butler A (2018). Integrating single-cell transcriptomic data across different conditions, technologies, and species. Nat. Biotechnol..

[21] Newman AM (2019). Determining cell type abundance and expression from bulk tissues with digital cytometry. Nat. Biotechnol..

[22] Malta TM (2018). Machine learning identifies stemness features associated with oncogenic dedifferentiation. Cell.

[23] Kupfer DM (2013). Microarray characterization of gene expression changes in blood during acute ethanol exposure. BMC Med. Genom..

[24] Chen J, Bardes EE, Aronow BJ, Jegga AG (2009). ToppGene suite for gene list enrichment analysis and candidate gene prioritization. Nucleic Acids Res..

[25] Cancer Genome Atlas Research, N (2013). The cancer genome atlas pan-cancer analysis project. Nat. Genet..

[26] Donovan MKR, D'Antonio-Chronowska A, D'Antonio M, Frazer KA (2020). Cellular deconvolution of GTEx tissues powers discovery of disease and cell-type associated regulatory variants. Nat. Commun..

[27] Duraturo F, Liccardo R, De Rosa M, Izzo P (2019). Genetics, diagnosis and treatment of Lynch syndrome: old lessons and current challenges. Oncol. Lett..

[28] Gehart H (2019). Identification of enteroendocrine regulators by real-time single-cell differentiation mapping. Cell.

[29] Cheng X (2019). Therapeutic potential of targeting the Wnt/beta-catenin signaling pathway in colorectal cancer. Biomed. Pharmacother..

[30] Brozinsky S, Fani K, Grosberg SJ, Wapnick S (1978). Alcohol ingestion-induced changes in the human rectal mucosa: light and electron microscopic studies. Dis. Colon Rectum.

[31] Shao T (2018). Intestinal HIF-1alpha deletion exacerbates alcoholic liver disease by inducing intestinal dysbiosis and barrier dysfunction. J. Hepatol..

[32] Simanowski UA (2001). Increased rectal cell proliferation following alcohol abuse. Gut.

[33] Muller MF, Zhou Y, Adams DJ, Arends MJ (2017). Effects of long-term ethanol consumption and Aldh1b1 depletion on intestinal tumourigenesis in mice. J. Pathol..

[34] Finch AJ (2009). Acute overexpression of Myc in intestinal epithelium recapitulates some but not all the changes elicited by Wnt/beta-catenin pathway activation. Mol. Cell. Biol..

[35] van de Wetering M (2002). The beta-catenin/TCF-4 complex imposes a crypt progenitor phenotype on colorectal cancer cells. Cell.

[36] Bras-Pereira C, Moreno E (2018). Mechanical cell competition. Curr. Opin. Cell Biol..

[37] Papapietro O (2013). R-spondin 2 signalling mediates susceptibility to fatal infectious diarrhoea. Nat. Commun..

[38] Hicks SD, Middleton FA, Miller MW (2010). Ethanol-induced methylation of cell cycle genes in neural stem cells. J. Neurochem..

[39] Siegenthaler JA, Miller MW (2005). Transforming growth factor beta 1 promotes cell cycle exit through the cyclin-dependent kinase inhibitor p21 in the developing cerebral cortex. J. Neurosci..

[40] Garaycoechea JI (2018). Alcohol and endogenous aldehydes damage chromosomes and mutate stem cells. Nature.

[41] Di Rocco G, Baldari S, Pani G, Toietta G (2019). Stem cells under the influence of alcohol: effects of ethanol consumption on stem/progenitor cells. Cell. Mol. Life Sci..

[42] Khacho M, Slack RS (2018). Mitochondrial and reactive oxygen species signaling coordinate stem cell fate decisions and life long maintenance. Antioxid. Redox Signal..

[43] Forsyth CB (2010). Alcohol stimulates activation of Snail, epidermal growth factor receptor signaling, and biomarkers of epithelial-mesenchymal transition in colon and breast cancer cells. Alcohol. Clin. Exp. Res..

[44] Park SC (2012). Ethanol-induced DNA damage and repair-related molecules in human intestinal epithelial Caco-2 cells. Mol. Med. Rep..

[45] Swanson G (2011). Role of intestinal circadian genes in alcohol-induced gut leakiness. Alcohol. Clin. Exp. Res..

[46] Lu R (2017). Alcohol injury damages intestinal stem cells. Alcohol. Clin. Exp. Res..

[47] Tang YM (2015). The role of miR-212 and iNOS in alcohol-induced intestinal barrier dysfunction and steatohepatitis. Alcohol. Clin. Exp. Res..

[48] Xu L, Lin W, Wen L, Li G (2019). Lgr5 in cancer biology: functional identification of Lgr5 in cancer progression and potential opportunities for novel therapy. Stem Cell Res. Ther..

[49] Xu M (2016). Role of MCP-1 in alcohol-induced aggressiveness of colorectal cancer cells. Mol. Carcinog..

[50] Neal JT (2018). Organoid modeling of the tumor immune microenvironment. Cell.

[51] Noel G (2017). A primary human macrophage-enteroid co-culture model to investigate mucosal gut physiology and host-pathogen interactions. Sci. Rep..

[52] Wang S (2012). Ethanol promotes mammary tumor growth and angiogenesis: the involvement of chemoattractant factor MCP-1. Breast Cancer Res. Treat..

[53] Dobin A (2013). STAR: ultrafast universal RNA-seq aligner. Bioinformatics.

[54] Zhou FC (2011). Alcohol alters DNA methylation patterns and inhibits neural stem cell differentiation. Alcohol. Clin. Exp. Res..

[55] R Core Team. *R: A Language and Environment for Statistical Computing* (R Foundation for Statistical Computing, Vienna, Austria, 2019).

[56] Love MI, Huber W, Anders S (2014). Moderated estimation of fold change and dispersion for RNA-seq data with DESeq2. Genome Biol..

[57] Soneson C, Love MI, Robinson MD (2015). Differential analyses for RNA-seq: transcript-level estimates improve gene-level inferences. F1000Res.

[58] Blighe, K., Rana, S. & Lewis, M. EnhancedVolcano: Publication-ready volcano plots with enhanced colouring and labeling. https://github.com/kevinblighe/EnhancedVolcano (2018).

[59] Ritchie ME (2015). limma powers differential expression analyses for RNA-sequencing and microarray studies. Nucleic Acids Res..

[60] Colaprico A (2016). TCGAbiolinks: an R/Bioconductor package for integrative analysis of TCGA data. Nucleic Acids Res..

