# Supplementary material for: Ethanol exposure drives colon location specific cell composition changes in a normal colon crypt 3D organoid model
Source: Sci Rep. 2021 Jan 11;11:432. doi: 10.1038/s41598-020-80240-1 (PMC7801615; doi:10.1038/s41598-020-80240-1)
Supplement: Supplementary file 1 — Supplementary Information. [file 41598_2020_80240_MOESM1_ESM.pdf]

Ethanol exposure drives colon location specific cell composition changes in a normal colon crypt 3D organoid model

Cell-specific response to ethanol in organoids

Matthew Devall[1], Sarah J Plummer[1], Jennifer Bryant[1], Lucas T Jennelle[1], Stephen Eaton[1], Christopher H Dampier[1,2], Jeroen Huyghe[4], Ulrike Peters[4], Steven M Powell[3], and Graham Casey[1*]

[1] Center for Public Health Genomics, Department of Public Health Sciences, University of Virginia, Charlottesville, VA, USA;

[2] Department of Surgery, Center for Public Health Genomics, University of Virginia, Charlottesville, VA, USA;

[3] Digestive Health Center, University of Virginia, Charlottesville, VA, USA

[4] Public Health Sciences Division, Fred Hutchinson Cancer Center Research Institute, Seattle, WA, USA

*gc8r@virginia.edu

Supplementary Table 1: Summary of demographic information provided for individual organoid pairs.

Pair	Sex	Age	Smoking Status	Location	BMI[#]	Polyp[+]
1	F	22	Never	Left	26.4	No
2	M	34	Former	Right	27.6	No
3	M	66	Never	Right	22.96	No
4	F	61	Current	Left	19.7	No
5	M	59	Never	Right	35.99	No
6	M	53	Never	Right	50.4	No
7	F	71	Never	Right	22.11	No
8	M	60	Never	Left	31.97	Yes
9	F	58	Never	Right	24.71	No
10	M	64	Never	Right	27.78	No
11	F	64	Never	Left	38.36	No
12	M	54	Never	Right	25.09	Yes
13	F	74	Never	Left	29.31	Yes
14	M	56	Former	Left	22.93	No
15	F	67	Former	Left	28.96	No
16	M	73	Never	Left	29.8	Yes
17	M	34	Never	Left	45	No
18	F	64	Never	Left	32.5	No
19	M	61	Current	Right	28	No
20	F	50	Former	Left	27.6	No
21	M	63	Current	Right	24.21	No
22	M	65	Never	Right	33	Yes
23	F	62	Never	Left	23.8	No
24	M	68	Current	Left	24.48	No
25	F	70	Never	Right	43	Yes
26	F	70	Never	Right	25.06	No
27	F	52	Current	Right	24.62	No
28	F	52	Current	Right	20.59	No
29	F	50	Never	Left	41.27	No
30	F	47	Never	Right	25.02	No
31	M	59	Never	Right	NA[*]	Yes
32	F	53	Never	Left	30.38	No
33	F	53	Never	Left	27.79	No
34	M	32	Never	Right	22.53	No

[#] BMI: body mass index
[+] Polyp: presence of three or more tubular adenomas at the time of colonoscopy
* NA: data not available.

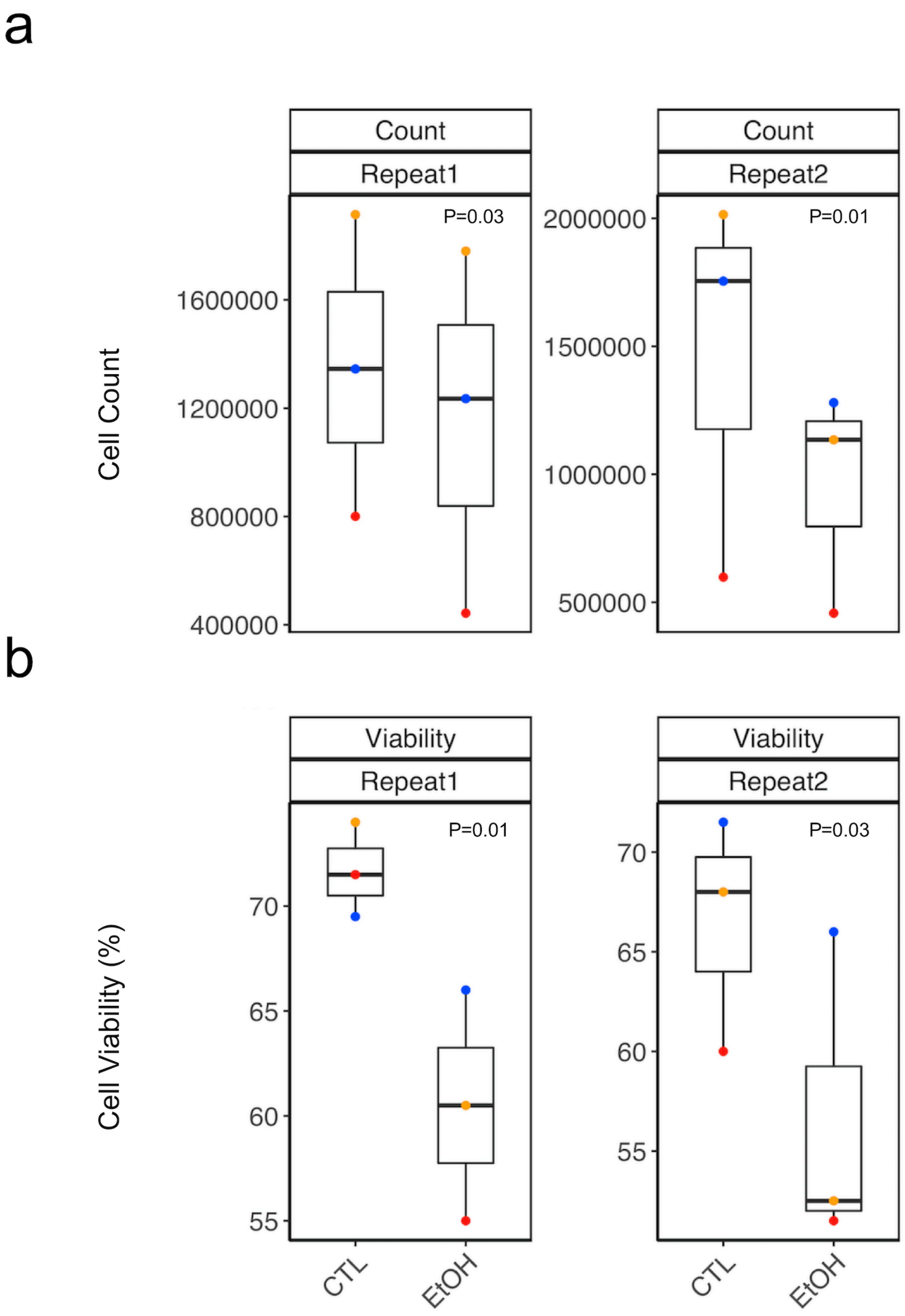

Supplementary Figure 1: Cell proliferation and viability analysis of left colon organoids (n=3). Colored points highlight the paired relationship between biological replicates. **A)** Cell counts were taken from two technical repeats and showed a consistent decrease in cell number in ethanol treated pairs. **B)** Cell viability measures were determined for each replicate. Viability was decreased in both technical replicates of the analysis.

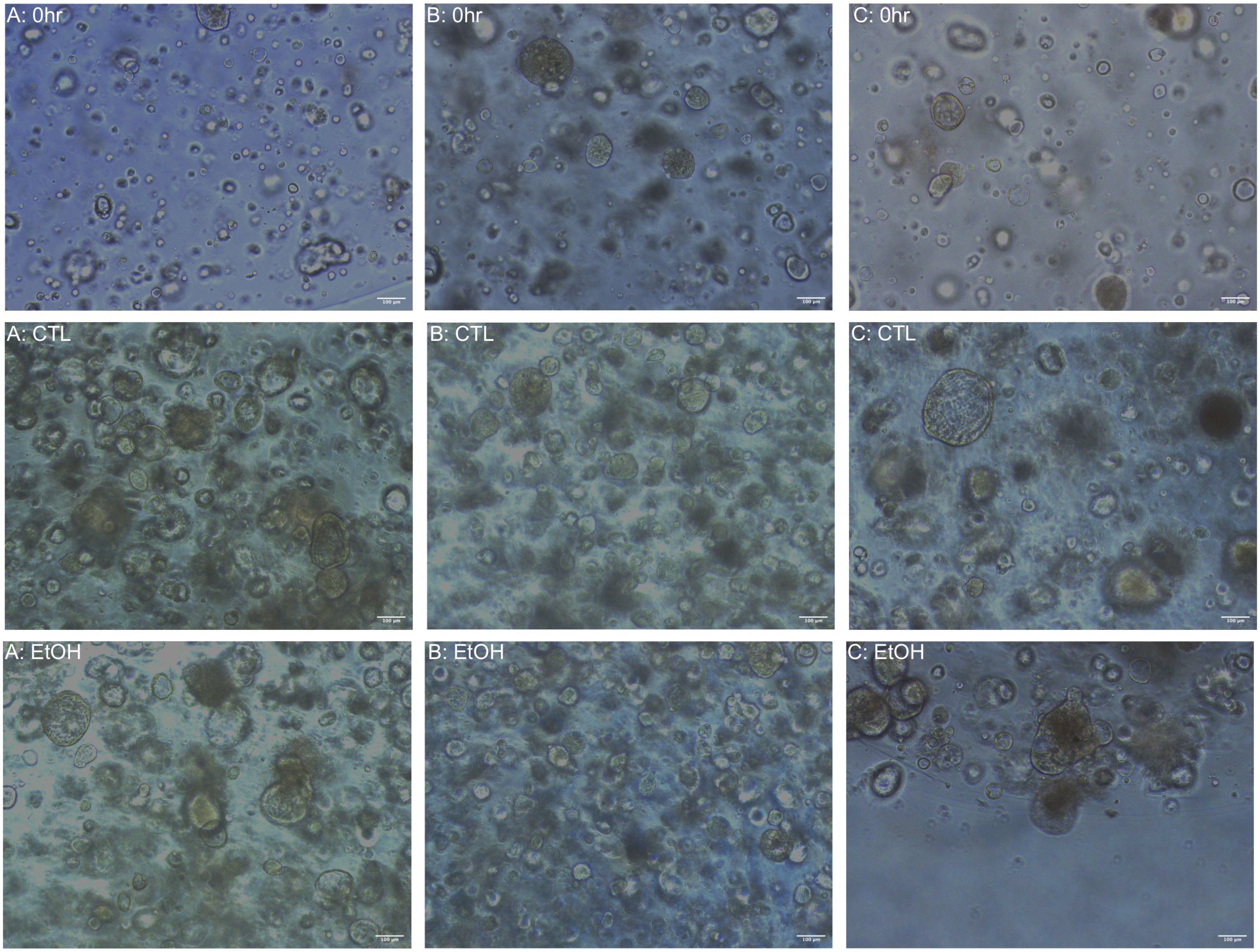

Supplementary Figure 2: Brightfield microscopic images (10x) of three colon organoid lines (A, B and C) derived from left colon organoids, prior (top) and following treatment to vehicle control (middle) and ethanol (bottom). Scale was set to 100 μm.

Supplementary Table 2: Summary of significant apoptosis-related gene expression in ethanol-treated colon organoids. Fold changes greater than one correspond to increase expression in ethanol treated organoids.

Ensembl ID	HGNC	Mean	FC	Stat	*P* value	FDR
ENSG00000055163	CYFIP2	1995	1.134	5.078	3.82E-07	1.95E-04
ENSG00000019582	CD74	2786	1.095	4.033	5.50E-05	3.90E-03
ENSG00000087586	AURKA	2379	1.088	4.013	5.99E-05	4.10E-03
ENSG00000067057	PFKP	13681	0.925	-3.938	8.20E-05	4.89E-03
ENSG00000101004	NINL	247	1.115	3.203	1.36E-03	0.025
ENSG00000164024	METAP1	4781	1.036	2.973	2.95E-03	0.038
ENSG00000206503	HLA-A	72443	0.946	-2.745	6.06E-03	0.058
ENSG00000087088	BAX	5493	1.036	2.682	7.31E-03	0.065
ENSG00000205581	HMGN1	11755	1.030	2.669	7.60E-03	0.067
ENSG00000134291	TMEM106C	7649	1.038	2.639	8.31E-03	0.070
ENSG00000242802	AP5Z1	1135	0.943	-2.631	8.51E-03	0.071
ENSG00000137752	CASP1	60	1.255	2.626	8.63E-03	0.072
ENSG00000105552	BCAT2	1330	1.049	2.487	0.013	0.091
ENSG00000155561	NUP205	3961	1.052	2.456	0.014	0.095

Supplementary Table 3: Validation of gene expression differences between ethanol and vehicle treated colon organoids. Fold changes greater than one correspond to increase expression in ethanol treated organoids

			Validation (n=6)		RNA-seq (n=34)	
**HGNC**	**Assay**	**Reason for Selection**	**FC**	***P* value**	**FC**	**FDR**
FSTL3	Hs00610505_m1	Novel to colon	0.717	6.72E-07	0.866	1.80E-07
FXYD3	Hs00254211_m1	Novel to colon	0.671	1.84E-06	0.910	0.014
HLA-G	Hs00365950_g1	CRC GWAS	0.726	9.96E-06	0.807	4.28E-04
MSLN	Hs00245879_m1	Novel	0.757	5.23E-05	0.895	1.10E-06
TMSB10	Hs00363670_m1	Novel for colon	0.763	6.13E-05	0.943	6.37E-03
CLIC3	Hs00362166_g1	Novel	0.684	1.52E-04	0.790	2.31E-05
DKK3	Hs00247426_m1	Novel for colon	1.195	6.70E-03	1.243	3.05E-05
RBBP7	Hs00171476_m1	Novel for colon	0.947	0.095	1.045	0.045
ALDH7A1	Hs00609622_m1	CRC GWAS	0.926	0.233	1.091	6.02E-05
SCG5	Hs00161638_m1	CRC GWAS	0.935	0.379	1.104	6.29E-03
POLD3	Hs01075255_m1	CRC GWAS	0.954	0.513	1.070	0.037

Supplementary Table 4: Differential expression results for left and right colon organoids exposed to ethanol following adjustment for cell composition. Fold changes greater than one correspond to increase expression in ethanol treated organoids.

		Left Colon Organoid					Right Colon Organoid				
**Ensembl ID**	**HGNC**	**Mean**	**FC**	**Stat**	***P* value**	**FDR**	**Mean**	**FC**	**Stat**	***P* value**	**FDR**
ENSG00000151651	ADAM8	1561	0.838	-5.958	2.55E-09	2.90E-05	2511	0.952	-1.252	0.210	0.872
ENSG00000143416	SELENBP1	2492	1.190	5.551	2.83E-08	1.61E-04	1609	1.040	1.156	0.248	0.886
ENSG00000113739	STC2	1403	0.815	-5.254	1.49E-07	5.65E-04	1759	0.972	-0.729	0.466	0.937
ENSG00000141449	GREB1L	2083	0.890	-4.708	2.50E-06	4.79E-03	2361	0.965	-1.598	0.110	0.822
ENSG00000167601	AXL	1132	0.838	-4.758	1.95E-06	4.79E-03	2136	1.042	1.175	0.240	0.880
ENSG00000091138	SLC26A3	16356	1.359	4.706	2.53E-06	4.79E-03	6599	1.050	0.724	0.469	0.937
ENSG00000176945	MUC20	6668	0.886	-4.530	5.91E-06	5.09E-03	7044	0.943	-1.975	0.048	0.767
ENSG00000122176	FMOD	607	1.254	4.530	5.91E-06	5.09E-03	308	1.162	1.452	0.146	0.842
ENSG00000117525	F3	13880	0.905	-4.547	5.44E-06	5.09E-03	16274	0.973	-1.330	0.184	0.858
ENSG00000243955	GSTA1	463	1.289	4.587	4.50E-06	5.09E-03	348	1.082	1.205	0.228	0.878
ENSG00000241635	UGT1A1	17943	1.092	4.525	6.05E-06	5.09E-03	12868	1.033	1.161	0.246	0.885
ENSG00000244474	UGT1A4	14625	1.089	4.489	7.16E-06	5.09E-03	10992	1.028	1.010	0.313	0.906
ENSG00000240224	UGT1A5	14502	1.090	4.500	6.78E-06	5.09E-03	10888	1.027	0.983	0.326	0.909
ENSG00000241119	UGT1A9	14484	1.090	4.516	6.31E-06	5.09E-03	10875	1.027	0.975	0.329	0.909
ENSG00000243135	UGT1A3	14514	1.090	4.531	5.88E-06	5.09E-03	10881	1.027	0.957	0.339	0.912
ENSG00000244122	UGT1A7	14585	1.089	4.499	6.82E-06	5.09E-03	10966	1.026	0.928	0.354	0.915
ENSG00000242366	UGT1A8	14872	1.089	4.418	9.98E-06	0.007	11244	1.029	1.038	0.299	0.900
ENSG00000136048	DRAM1	851	1.177	4.371	1.24E-05	0.008	685	1.051	1.306	0.192	0.862
ENSG00000181634	TNFSF15	3928	1.112	4.330	1.49E-05	0.009	3587	0.988	-0.526	0.599	0.961
ENSG00000172137	CALB2	1396	0.841	-4.239	2.25E-05	0.013	2193	0.998	-0.081	0.936	0.994
ENSG00000050165	DKK3	321	1.238	4.205	2.61E-05	0.014	536	1.073	1.181	0.238	0.880
ENSG00000167165	UGT1A6	16953	1.081	4.157	3.22E-05	0.015	12979	1.032	1.142	0.254	0.886
ENSG00000165092	ALDH1A1	7969	1.126	4.163	3.14E-05	0.015	8013	1.033	1.016	0.309	0.903
ENSG00000180914	OXTR	550	0.832	-4.164	3.13E-05	0.015	544	0.955	-0.799	0.425	0.930
ENSG00000242515	UGT1A10	20112	1.080	4.176	2.97E-05	0.015	15614	1.021	0.768	0.442	0.934
ENSG00000149925	ALDOA	100572	0.893	-4.134	3.57E-05	0.016	115742	1.004	0.138	0.890	0.990
ENSG00000085662	AKR1B1	2517	1.106	4.102	4.10E-05	0.017	2218	1.020	0.797	0.425	0.930
ENSG00000101298	SNPH	286	1.263	4.069	4.72E-05	0.018	208	1.117	1.586	0.113	0.823
ENSG00000103257	SLC7A5	5622	0.904	-4.075	4.60E-05	0.018	6932	1.025	1.029	0.303	0.901
ENSG00000146674	IGFBP3	29856	0.810	-4.056	5.00E-05	0.019	38601	0.914	-3.312	9.25E-04	0.605
ENSG00000102854	MSLN	5923	0.900	-3.994	6.49E-05	0.024	7022	0.955	-1.944	0.052	0.779
ENSG00000058668	ATP2B4	4291	0.889	-3.955	7.64E-05	0.027	6330	0.951	-2.086	0.037	0.740
ENSG00000070669	ASNS	3714	0.882	-3.939	8.20E-05	0.028	5501	0.982	-0.597	0.551	0.954

ENSG00000012779	ALOX5	1716	0.869	-3.919	8.89E-05	0.030	2027	0.926	-1.028	0.304	0.901
ENSG00000173597	SULT1B1	2453	1.153	3.905	9.44E-05	0.031	1433	1.044	0.584	0.559	0.956
ENSG00000152256	PDK1	3069	0.874	-3.896	9.80E-05	0.031	3792	0.929	-2.395	0.017	0.716
ENSG00000105220	GPI	23348	0.914	-3.889	1.01E-04	0.031	23392	1.039	1.549	0.121	0.828
ENSG00000149809	TM7SF2	1531	1.108	3.858	1.14E-04	0.034	1266	1.060	1.863	0.063	0.787
ENSG00000167755	KLK6	25118	0.905	-3.854	1.16E-04	0.034	34577	1.032	1.286	0.198	0.866
ENSG00000285043		89747	0.899	-3.825	1.31E-04	0.037	103187	1.005	0.192	0.848	0.987
ENSG00000104267	CA2	5997	1.131	3.786	1.53E-04	0.042	4078	1.000	-0.003	0.998	1.000
ENSG00000167772	ANGPTL4	2594	0.894	-3.766	1.66E-04	0.043	3533	0.907	-1.532	0.126	0.828
ENSG00000003436	TFPI	1710	0.891	-3.769	1.64E-04	0.043	2871	0.961	-1.352	0.176	0.858
ENSG00000163975	MELTF	3964	0.915	-3.774	1.61E-04	0.043	4918	0.996	-0.184	0.854	0.987
ENSG00000187091	PLCD1	994	1.132	3.743	1.82E-04	0.045	987	1.071	1.717	0.086	0.807
ENSG00000185432	METTL7A	787	1.187	3.745	1.80E-04	0.045	926	1.059	1.321	0.186	0.859
ENSG00000099834	CDHR5	8821	0.914	-3.733	1.89E-04	0.046	8049	1.006	0.174	0.862	0.987
ENSG00000204128	C2orf72	4463	1.092	3.723	1.97E-04	0.047	3037	1.040	1.692	0.091	0.815
ENSG00000160213	CSTB	17415	0.925	-3.692	2.22E-04	0.052	17636	0.941	-2.386	0.017	0.717
ENSG00000133742	CA1	1009	1.558	3.660	2.52E-04	0.057	277	0.969	-0.203	0.839	0.987
ENSG00000073849	ST6GAL1	2457	1.104	3.634	2.79E-04	0.059	1815	1.078	2.238	0.025	0.718
ENSG00000162366	PDZK1IP1	3623	0.877	-3.647	2.65E-04	0.059	2927	0.938	-1.794	0.073	0.801
ENSG00000003402	CFLAR	8783	0.937	-3.635	2.78E-04	0.059	9726	0.966	-1.756	0.079	0.806
ENSG00000137501	SYTL2	8221	1.097	3.642	2.71E-04	0.059	6033	1.004	0.170	0.865	0.987
ENSG00000136155	SCEL	1026	0.875	-3.590	3.31E-04	0.067	1634	0.895	-2.682	7.33E-03	0.680
ENSG00000100003	SEC14L2	3277	0.912	-3.585	3.37E-04	0.067	3818	0.951	-2.247	0.025	0.718
ENSG00000123933	MXD4	2563	1.098	3.588	3.33E-04	0.067	2365	0.994	-0.229	0.819	0.986
ENSG00000232434	AJM1	693	0.856	-3.565	3.64E-04	0.071	943	0.906	-2.257	0.024	0.718
ENSG00000170689	HOXB9	3750	1.084	3.523	4.27E-04	0.081	4441	1.043	1.826	0.068	0.793
ENSG00000197747	S100A10	50921	0.930	-3.512	4.45E-04	0.081	49635	0.956	-1.683	0.092	0.817
ENSG00000139567	ACVRL1	552	1.164	3.507	4.54E-04	0.081	310	1.111	1.545	0.122	0.828
ENSG00000095321	CRAT	2311	0.915	-3.516	4.39E-04	0.081	2702	0.966	-1.428	0.153	0.849
ENSG00000173559	NABP1	2257	0.914	-3.510	4.48E-04	0.081	2378	0.958	-1.341	0.180	0.858
ENSG00000070540	WIPI1	866	1.127	3.518	4.34E-04	0.081	775	1.004	0.112	0.911	0.992
ENSG00000134333	LDHA	120783	0.898	-3.492	4.79E-04	0.084	118933	0.959	-1.603	0.109	0.822
ENSG00000164904	ALDH7A1	4670	1.091	3.477	5.08E-04	0.087	4726	1.024	1.146	0.252	0.886
ENSG00000188242		1429	1.129	3.443	5.74E-04	0.096	1003	1.109	2.146	0.032	0.722
ENSG00000128311	TST	12222	1.067	3.443	5.75E-04	0.096	10392	1.011	0.461	0.645	0.967
ENSG00000169583	CLIC3	357	0.875	-1.937	0.053	0.847	548	0.777	-4.901	9.54E-07	0.016
ENSG00000244257	PKD1P1	277	0.965	-0.539	0.590	0.999	386	0.818	-4.629	3.68E-06	0.030
ENSG00000105219	CNTD2	12	1.091	0.295	0.768	NA	33	0.468	-4.555	5.23E-06	0.030

Supplementary Table 5: Differential expression results for male and female colon organoids exposed to ethanol following adjustment for cell composition. Fold changes greater than one correspond to increase expression in ethanol treated organoids.

		**Male Colon Organoid**					**Female Colon Organoid**				
**Ensembl ID**	**HGNC**	**Mean**	**FC**	**Stat**	***P* value**	**FDR**	**Mean**	**FC**	**Stat**	***P* value**	**FDR**
ENSG00000153233	PTPRR	8724	-0.174	-5.178	2.24E-07	3.54E-03	8426	-0.036	-1.070	0.285	0.937
ENSG00000092068	SLC7A8	432	0.390	4.878	1.07E-06	5.66E-03	302	0.049	0.553	0.580	0.974
ENSG00000101188	NTSR1	654	-0.355	-4.879	1.07E-06	5.66E-03	664	-0.035	-0.225	0.822	0.997
ENSG00000103044	HAS3	3000	-0.170	-4.693	2.69E-06	0.011	2615	-0.042	-0.981	0.326	0.951
ENSG00000178828	RNF186	1908	-0.220	-4.360	1.30E-05	0.041	1518	-0.004	-0.071	0.944	1.000
ENSG00000100417	PMM1	1352	-0.194	-4.303	1.69E-05	0.044	1252	0.060	1.066	0.286	0.939
ENSG00000169583	CLIC3	489	-0.341	-4.249	2.15E-05	0.048	432	-0.274	-3.075	2.10E-03	0.323
ENSG00000175567	UCP2	991	0.226	4.172	3.02E-05	0.057	862	0.163	3.119	1.81E-03	0.292
ENSG00000247095	MIR210HG	2504	-0.204	-4.157	3.22E-05	0.057	2409	-0.054	-1.062	0.288	0.939
ENSG00000171056	SOX7	189	-0.526	-4.070	4.69E-05	0.067	218	-0.166	-1.439	0.150	0.889
ENSG00000105219	CNTD2	29	-1.007	-4.087	4.36E-05	0.067	18	0.194	0.530	0.596	NA
ENSG00000006747	SCIN	2859	-0.124	-4.039	5.38E-05	0.071	2821	-0.087	-1.849	0.064	0.801
ENSG00000058668	ATP2B4	5407	-0.145	-3.978	6.94E-05	0.079	5332	-0.121	-3.273	1.06E-03	0.221
ENSG00000183873	SCN5A	236	-0.922	-3.976	7.02E-05	0.079	183	-0.197	-1.478	0.139	0.885
ENSG00000102359	SRPX2	221	-0.388	-3.899	9.66E-05	0.098	204	-0.324	-3.333	8.59E-04	0.217
ENSG00000173376	NDNF	263	-0.403	-3.892	9.96E-05	0.098	306	-0.018	-0.199	0.843	0.999
ENSG00000070404	FSTL3	2097	-0.147	-3.231	1.23E-03	0.330	2268	-0.214	-4.277	1.89E-05	0.029
ENSG00000105971	CAV2	5924	-0.112	-3.178	1.48E-03	0.330	6135	-0.168	-4.160	3.18E-05	0.030
ENSG00000102854	MSLN	6339	-0.128	-3.152	1.62E-03	0.331	6618	-0.143	-4.007	6.15E-05	0.047
ENSG00000146674	IGFBP3	35984	-0.209	-3.007	2.64E-03	0.366	33205	-0.196	-5.930	3.03E-09	3.06E-05
ENSG00000232434	AJM1	918	-0.142	-2.499	0.012	0.500	750	-0.272	-4.187	2.82E-05	0.030
ENSG00000151651	ADAM8	2302	-0.131	-2.450	0.014	0.521	1872	-0.253	-4.303	1.69E-05	0.029
ENSG00000167757	KLK11	2561	-0.122	-2.375	0.018	0.544	3036	-0.171	-3.960	7.50E-05	0.053
ENSG00000243955	GSTA1	425	0.222	2.257	0.024	0.581	383	0.324	3.882	1.04E-04	0.070
ENSG00000152256	PDK1	3606	-0.154	-2.258	0.024	0.581	3321	-0.133	-3.813	1.37E-04	0.077
ENSG00000107159	CA9	16535	-0.452	-2.246	0.025	0.581	14991	-0.248	-5.845	5.06E-09	3.06E-05
ENSG00000136048	DRAM1	759	0.120	2.162	0.031	0.623	766	0.196	4.134	3.57E-05	0.031
ENSG00000130822	PNCK	471	-0.513	-1.982	0.048	0.686	470	-0.416	-4.213	2.52E-05	0.030
ENSG00000065618	COL17A1	61322	-0.164	-1.704	0.088	0.766	51983	-0.133	-3.829	1.29E-04	0.077
ENSG00000090013	BLVRB	3305	-0.051	-1.680	0.093	0.775	2753	-0.213	-3.808	1.40E-04	0.077
ENSG00000136155	SCEL	1557	-0.139	-1.629	0.103	0.787	1175	-0.206	-4.500	6.79E-06	0.014
ENSG00000249948	GBA3	443	0.262	1.428	0.153	0.837	263	0.427	3.703	2.13E-04	0.099
ENSG00000142623	PADI1	1035	-0.091	-1.380	0.168	0.844	1254	-0.319	-4.494	6.98E-06	0.014

ENSG00000090382	LYZ	88052	0.082	1.362	0.173	0.848	114891	0.141	4.816	1.46E-06	5.90E-03
ENSG00000172137	CALB2	2044	-0.108	-1.307	0.191	0.857	1632	-0.166	-3.719	2.00E-04	0.097
ENSG00000113739	STC2	1801	-0.073	-0.847	0.397	0.943	1420	-0.218	-4.218	2.47E-05	0.030
ENSG00000012779	ALOX5	2004	-0.095	-0.770	0.441	0.949	1779	-0.233	-4.072	4.66E-05	0.038
ENSG00000117525	F3	14450	-0.026	-0.672	0.502	0.960	15649	-0.136	-3.757	1.72E-04	0.089
ENSG00000095321	CRAT	2552	-0.019	-0.616	0.538	0.962	2487	-0.170	-4.590	4.43E-06	0.013
ENSG00000167759	KLK13	217	-0.047	-0.496	0.620	0.972	279	-0.419	-4.160	3.19E-05	0.030
ENSG00000106003	LFNG	3198	0.016	0.473	0.636	0.977	2807	0.165	3.750	1.77E-04	0.089
ENSG00000070669	ASNS	5218	-0.006	-0.166	0.868	0.996	4203	-0.182	-3.850	1.18E-04	0.075

Supplementary Table 6: Cell-type agnostic DEGs identified for ethanol treatment that were significant following cell type adjustment. Fold change greater than one indicates increased expression versus vehicle control organoids. TRUE indicates validation in blood analysis.

Ensembl ID	HGNC Symbol	Mean	FC	Test Statistic	*P* Value	FDR	Validated in Blood Analysis
ENSG00000143416	SELENBP1	2028	1.114	4.347	1.38E-05	8.80E-03	TRUE
ENSG00000066230	SLC9A3	2509	1.130	4.303	1.68E-05	9.32E-03	TRUE
ENSG00000204128	C2orf72	3713	1.062	4.064	4.83E-05	0.017	TRUE
ENSG00000188242		1205	1.126	3.943	8.04E-05	0.022	
ENSG00000134243	SORT1	6498	1.047	3.917	8.96E-05	0.022	
ENSG00000112977	DAP	9076	1.045	3.865	1.11E-04	0.023	
ENSG00000161970	RPL26	43994	0.933	-3.788	1.52E-04	0.026	
ENSG00000134419	RPS15A	31457	0.939	-3.749	1.78E-04	0.027	TRUE
ENSG00000166348	USP54	2406	1.054	3.661	2.52E-04	0.034	
ENSG00000112343	TRIM38	1772	1.049	3.627	2.87E-04	0.036	
ENSG00000129353	SLC44A2	4061	1.049	3.573	3.53E-04	0.043	
ENSG00000163590	PPM1L	1028	1.072	3.564	3.65E-04	0.043	
ENSG00000139567	ACVRL1	424	1.146	3.495	4.75E-04	0.049	TRUE
ENSG00000081181	ARG2	573	1.137	3.497	4.70E-04	0.049	
ENSG00000124574	ABCC10	1753	1.060	3.429	6.05E-04	0.057	
ENSG00000244257	PKD1P1	335	0.865	-3.425	6.14E-04	0.057	
ENSG00000116133	DHCR24	22789	1.042	3.393	6.92E-04	0.061	
ENSG00000172137	CALB2	1818	0.908	-3.390	6.99E-04	0.061	
ENSG00000279861		889	1.081	3.371	7.48E-04	0.063	
ENSG00000129116	PALLD	3053	1.075	3.366	7.62E-04	0.063	
ENSG00000131016	AKAP12	3908	0.917	-3.338	8.44E-04	0.066	TRUE
ENSG00000066739	ATG2B	1545	1.055	3.322	8.95E-04	0.067	
ENSG00000135540	NHSL1	2052	1.059	3.319	9.05E-04	0.067	TRUE
ENSG00000176171	BNIP3	5384	0.901	-3.283	1.03E-03	0.072	TRUE
ENSG00000175793	SFN	9511	1.045	3.268	1.08E-03	0.074	TRUE
ENSG00000189319	FAM53B	3012	1.064	3.260	1.11E-03	0.075	
ENSG00000241635	UGT1A1	15277	1.062	3.261	1.11E-03	0.075	
ENSG00000142039	CCDC97	1217	1.061	3.257	1.13E-03	0.076	
ENSG00000073969	NSF	2523	1.046	3.253	1.14E-03	0.076	
ENSG00000244474	UGT1A4	12718	1.059	3.247	1.17E-03	0.077	
ENSG00000167165	UGT1A6	14867	1.058	3.248	1.16E-03	0.077	
ENSG00000103005	USB1	1827	1.061	3.227	1.25E-03	0.079	
ENSG00000240224	UGT1A5	12605	1.059	3.213	1.31E-03	0.081	
ENSG00000241119	UGT1A9	12590	1.059	3.205	1.35E-03	0.082	
ENSG00000243135	UGT1A3	12607	1.059	3.207	1.34E-03	0.082	
ENSG00000242366	UGT1A8	12968	1.059	3.194	1.41E-03	0.083	
ENSG00000148337	CIZ1	2832	1.045	3.183	1.46E-03	0.085	
ENSG00000244122	UGT1A7	12686	1.058	3.169	1.53E-03	0.087	
ENSG00000129667	RHBDF2	1399	1.059	3.161	1.57E-03	0.087	
ENSG00000115457	IGFBP2	4743	0.941	-3.159	1.58E-03	0.087	
ENSG00000139531	SUOX	1077	1.076	3.143	1.67E-03	0.091	
ENSG00000265972	TXNIP	38423	0.921	-3.142	1.68E-03	0.091	
ENSG00000135046	ANXA1	18618	0.953	-3.135	1.72E-03	0.091	TRUE
ENSG00000179454	KLHL28	1291	0.937	-3.135	1.72E-03	0.091	TRUE
ENSG00000109475	RPL34	23276	0.949	-3.107	1.89E-03	0.094	TRUE
ENSG00000163975	MELTF	4471	0.954	-3.103	1.92E-03	0.094	

ENSG00000162415	ZSWIM5	577	1.080	3.092	1.99E-03	0.095	
ENSG00000171863	RPS7	35409	0.943	-3.090	2.00E-03	0.095	
ENSG00000255717	SNHG1	2019	0.943	-3.085	2.04E-03	0.095	
ENSG00000118369	USP35	493	1.077	3.086	2.03E-03	0.095	TRUE
ENSG00000170365	SMAD1	739	1.075	3.073	2.12E-03	0.097	
ENSG00000150093	ITGB1	48864	0.959	-3.073	2.12E-03	0.097	TRUE
ENSG00000267080	ASB16-AS1	253	1.111	3.062	2.20E-03	0.099	
ENSG00000130255	RPL36	16726	0.949	-3.061	2.20E-03	0.099	TRUE
ENSG00000197386	HTT	8272	1.035	3.059	2.22E-03	0.100	

Supplementary Table 7: TCGA-COAD DEGs that are also found to be differentially expressed in ethanol after adjusting for cell composition. Fold changes greater than 1 indicate increased expression in paired normal adjacent tissue versus tumor.

Ensembl ID	HGNC Symbol	Mean	FC	Test Statistic	*P* Value	FDR
ENSG00000120708	TGFBI	26503	12.871	-10.483	1.03E-25	4.78E-23
ENSG00000006118	TMEM132A	1087	6.502	-10.234	1.39E-24	5.96E-22
ENSG00000242366	UGT1A8	358	0.024	9.482	2.50E-21	6.62E-19
ENSG00000111110	PPM1H	1193	3.938	-8.062	7.47E-16	7.16E-14
ENSG00000113739	STC2	485	11.101	-8.014	1.11E-15	1.03E-13
ENSG00000146674	IGFBP3	4261	3.344	-8.005	1.20E-15	1.09E-13
ENSG00000107159	CA9	1257	27.630	-7.611	2.72E-14	1.96E-12
ENSG00000249948	GBA3	730	0.054	7.576	3.56E-14	2.49E-12
ENSG00000073737	DHRS9	3576	0.054	7.485	7.14E-14	4.64E-12
ENSG00000185432	METTL7A	5885	0.233	7.424	1.14E-13	7.07E-12
ENSG00000102359	SRPX2	867	13.074	-7.291	3.09E-13	1.69E-11
ENSG00000073734	ABCB11	123	0.034	7.286	3.20E-13	1.74E-11
ENSG00000100003	SEC14L2	212	5.513	-7.229	4.87E-13	2.60E-11
ENSG00000140450	ARRDC4	2278	0.300	7.007	2.43E-12	1.10E-10
ENSG00000070404	FSTL3	756	6.030	-6.914	4.71E-12	2.01E-10
ENSG00000091138	SLC26A3	84086	0.019	6.844	7.70E-12	3.12E-10
ENSG00000241635	UGT1A1	100	0.035	6.826	8.72E-12	3.47E-10
ENSG00000129455	KLK8	38	230.684	-6.798	1.06E-11	4.13E-10
ENSG00000187091	PLCD1	2142	0.289	6.663	2.68E-11	9.61E-10
ENSG00000140939	NOL3	769	2.747	-6.649	2.95E-11	1.05E-09
ENSG00000006747	SCIN	2331	0.134	6.433	1.25E-10	3.84E-09
ENSG00000153233	PTPRR	930	0.142	6.164	7.10E-10	1.82E-08
ENSG00000142089	IFITM3	17208	3.722	-6.075	1.24E-09	2.97E-08
ENSG00000143416	SELENBP1	32517	0.189	6.058	1.38E-09	3.27E-08
ENSG00000204387	SNHG32	2905	2.951	-6.042	1.53E-09	3.57E-08
ENSG00000204128	C2orf72	1645	0.312	5.959	2.55E-09	5.66E-08
ENSG00000170365	SMAD1	898	0.586	5.953	2.64E-09	5.84E-08
ENSG00000173083	HPSE	635	0.274	5.926	3.11E-09	6.76E-08
ENSG00000112715	VEGFA	4496	2.535	-5.891	3.85E-09	8.19E-08
ENSG00000204361	NXPE2	244	0.157	5.833	5.46E-09	1.12E-07
ENSG00000159423	ALDH4A1	866	3.752	-5.797	6.74E-09	1.34E-07
ENSG00000205336	ADGRG1	8568	2.843	-5.658	1.53E-08	2.81E-07
ENSG00000255717	SNHG1	1305	2.407	-5.451	5.02E-08	8.06E-07
ENSG00000118523	CCN2	4821	2.814	-5.423	5.87E-08	9.25E-07
ENSG00000172927	MYEOV	707	6.747	-5.396	6.80E-08	1.05E-06
ENSG00000129667	RHBDF2	1408	2.629	-5.298	1.17E-07	1.69E-06
ENSG00000100311	PDGFB	567	2.202	-5.255	1.48E-07	2.09E-06

ENSG00000112655	PTK7	3016	4.483	-5.251	1.51E-07	2.13E-06
ENSG00000162129	CLPB	658	2.218	-5.211	1.87E-07	2.57E-06
ENSG00000188242		6770	0.180	5.133	2.85E-07	3.72E-06
ENSG00000139629	GALNT6	2210	3.152	-5.030	4.89E-07	5.97E-06
ENSG00000090530	P3H2	992	0.228	5.001	5.69E-07	6.82E-06
ENSG00000089356	FXYD3	37303	0.359	4.974	6.57E-07	7.73E-06
ENSG00000151651	ADAM8	589	3.006	-4.933	8.11E-07	9.32E-06
ENSG00000096968	JAK2	706	0.381	4.863	1.16E-06	1.27E-05
ENSG00000112031	MTRF1L	387	0.499	4.814	1.48E-06	1.56E-05
ENSG00000167757	KLK11	351	6.667	-4.751	2.02E-06	2.04E-05
ENSG00000148180	GSN	30505	0.488	4.749	2.05E-06	2.06E-05
ENSG00000182541	LIMK2	3950	0.585	4.707	2.52E-06	2.48E-05
ENSG00000066230	SLC9A3	8161	0.121	4.700	2.61E-06	2.55E-05
ENSG00000182272	B4GALNT4	302	7.269	-4.643	3.44E-06	3.25E-05
ENSG00000174358	SLC6A19	2342	0.073	4.614	3.94E-06	3.67E-05
ENSG00000100097	LGALS1	8528	3.162	-4.588	4.47E-06	4.09E-05
ENSG00000149809	TM7SF2	844	2.541	-4.582	4.61E-06	4.21E-05
ENSG00000180861	LINC01559	2187	0.211	4.581	4.63E-06	4.22E-05
ENSG00000163975	MELTF	1774	3.566	-4.560	5.11E-06	4.58E-05
ENSG00000050165	DKK3	2309	2.385	-4.558	5.16E-06	4.61E-05
ENSG00000248905	FMN1	1409	0.388	4.558	5.17E-06	4.62E-05
ENSG00000197747	S100A10	18014	0.528	4.545	5.50E-06	4.87E-05
ENSG00000139567	ACVRL1	4986	0.390	4.529	5.92E-06	5.20E-05
ENSG00000102854	MSLN	1611	5.869	-4.454	8.41E-06	7.06E-05
ENSG00000143149	ALDH9A1	4064	0.691	4.393	1.12E-05	9.00E-05
ENSG00000169583	CLIC3	168	3.428	-4.350	1.36E-05	1.07E-04
ENSG00000118263	KLF7	764	2.611	-4.332	1.48E-05	1.14E-04
ENSG00000197766	CFD	3058	0.321	4.330	1.49E-05	1.15E-04
ENSG00000159335	PTMS	8450	2.293	-4.329	1.50E-05	1.16E-04
ENSG00000167644	C19orf33	2583	0.317	4.266	1.99E-05	1.47E-04
ENSG00000134240	HMGCS2	23627	0.187	4.203	2.63E-05	1.88E-04
ENSG00000130255	RPL36	23981	1.845	-4.184	2.86E-05	2.02E-04
ENSG00000118369	USP35	242	1.751	-4.152	3.30E-05	2.29E-04
ENSG00000073969	NSF	2420	0.662	4.127	3.67E-05	2.51E-04
ENSG00000112343	TRIM38	1312	0.599	4.110	3.96E-05	2.68E-04
ENSG00000136048	DRAM1	974	1.837	-4.097	4.19E-05	2.81E-04
ENSG00000163590	PPM1L	787	0.450	4.042	5.29E-05	3.43E-04
ENSG00000134243	SORT1	6322	0.561	4.012	6.02E-05	3.85E-04
ENSG00000124574	ABCC10	1460	1.804	-4.000	6.35E-05	4.02E-04
ENSG00000142156	COL6A1	14418	2.366	-3.943	8.03E-05	4.91E-04
ENSG00000139211	AMIGO2	834	2.797	-3.921	8.83E-05	5.33E-04
ENSG00000120885	CLU	7636	0.386	3.899	9.64E-05	5.76E-04
ENSG00000112304	ACOT13	1524	0.569	3.839	1.24E-04	7.10E-04
ENSG00000152256	PDK1	1096	0.594	3.776	1.59E-04	8.82E-04
ENSG00000241119	UGT1A9	4	0.139	3.743	1.82E-04	9.84E-04
ENSG00000172137	CALB2	322	0.320	3.741	1.83E-04	9.89E-04
ENSG00000143344	RGL1	875	0.528	3.725	1.95E-04	1.05E-03
ENSG00000003402	CFLAR	3984	0.679	3.691	2.24E-04	1.18E-03
ENSG00000142623	PADI1	47	3.948	-3.640	2.73E-04	1.39E-03
ENSG00000135540	NHSL1	3469	0.581	3.543	3.95E-04	1.90E-03
ENSG00000160211	G6PD	2088	1.750	-3.512	4.44E-04	2.09E-03
ENSG00000129353	SLC44A2	7059	0.671	3.488	4.87E-04	2.25E-03
ENSG00000065618	COL17A1	8294	0.312	3.467	5.27E-04	2.40E-03

ENSG00000117151	CTBS	968	0.525	3.431	6.02E-04	2.69E-03
ENSG00000186522	SEPTIN10	2553	0.616	3.416	6.36E-04	2.82E-03
ENSG00000134905	CARS2	2559	1.710	-3.414	6.40E-04	2.83E-03
ENSG00000188959	C9orf152	3119	0.605	3.365	7.65E-04	3.29E-03
ENSG00000243955	GSTA1	780	0.238	3.269	1.08E-03	4.41E-03
ENSG00000050426	LETMD1	2248	1.417	-3.237	1.21E-03	4.86E-03
ENSG00000148337	CIZ1	4833	1.467	-3.212	1.32E-03	5.23E-03
ENSG00000166959	MS4A8	931	0.344	3.204	1.36E-03	5.35E-03
ENSG00000072506	HSD17B10	4025	1.814	-3.183	1.46E-03	5.68E-03
ENSG00000280693	SH3PXD2A-AS1	113	2.848	-3.136	1.71E-03	6.48E-03
ENSG00000111206	FOXM1	1903	1.623	-3.094	1.98E-03	7.29E-03
ENSG00000133835	HSD17B4	5997	0.681	3.087	2.02E-03	7.44E-03
ENSG00000185305	ARL15	929	0.621	3.050	2.29E-03	8.27E-03
ENSG00000258818	RNASE4	134	0.488	3.041	2.36E-03	8.48E-03
ENSG00000081181	ARG2	178	2.047	-3.035	2.41E-03	8.63E-03
ENSG00000213213	CCDC183	115	1.960	-2.996	2.73E-03	9.61E-03
ENSG00000265972	TXNIP	33567	0.531	2.991	2.78E-03	9.75E-03
ENSG00000054179	ENTPD2	1195	2.040	-2.977	2.91E-03	0.010
ENSG00000021300	PLEKHB1	1587	2.097	-2.877	4.01E-03	0.013
ENSG00000279861		41	2.135	-2.869	4.12E-03	0.014
ENSG00000135108	FBXO21	2057	1.329	-2.857	4.28E-03	0.014
ENSG00000141449	GREB1L	30	2.718	-2.844	4.46E-03	0.014
ENSG00000006534	ALDH3B1	1475	1.682	-2.843	4.47E-03	0.015
ENSG00000171056	SOX7	107	0.610	2.803	5.06E-03	0.016
ENSG00000175793	SFN	9899	0.540	2.763	5.73E-03	0.018
ENSG00000179454	KLHL28	534	0.649	2.730	6.33E-03	0.019
ENSG00000214193	SH3D21	402	1.870	-2.622	8.74E-03	0.025
ENSG00000113761	ZNF346	357	1.302	-2.620	8.78E-03	0.025
ENSG00000101474	APMAP	5586	1.609	-2.596	9.42E-03	0.027
ENSG00000101347	SAMHD1	3465	0.694	2.589	9.62E-03	0.027
ENSG00000107331	ABCA2	3548	1.807	-2.527	0.011	0.032
ENSG00000258791	LINC00520	11	0.337	2.511	0.012	0.033
ENSG00000057252	SOAT1	1455	0.634	2.501	0.012	0.034
ENSG00000185176	AQP12B	13	0.364	2.478	0.013	0.035
ENSG00000019582	CD74	53974	0.615	2.438	0.015	0.039
ENSG00000101213	PTK6	3691	0.611	2.373	0.018	0.045
ENSG00000143621	ILF2	6545	1.226	-2.275	0.023	0.056
ENSG00000109861	CTSC	6894	0.763	2.255	0.024	0.058
ENSG00000180902	D2HGDH	1591	1.694	-2.235	0.025	0.061
ENSG00000103260	METRN	790	1.912	-2.205	0.027	0.064
ENSG00000092964	DPYSL2	3755	1.551	-2.188	0.029	0.067
ENSG00000167772	ANGPTL4	399	1.769	-2.164	0.030	0.070
ENSG00000232434	AJM1	427	1.570	-2.161	0.031	0.071
ENSG00000162769	FLVCR1	1026	1.414	-2.145	0.032	0.073
ENSG00000066739	ATG2B	1238	0.701	2.105	0.035	0.079
ENSG00000058668	ATP2B4	6230	0.635	2.065	0.039	0.086
ENSG00000164684	ZNF704	1568	0.688	2.050	0.040	0.088
ENSG00000131844	MCCC2	4248	0.822	1.987	0.047	0.100
